# Nanofiltration Mixed Matrix Membranes from Cellulose Modified with Zn-Based Metal–Organic Frameworks for the Enhanced Water Treatment from Heavy Metal Ions

**DOI:** 10.3390/polym15061341

**Published:** 2023-03-07

**Authors:** Mariia Dmitrenko, Anna Kuzminova, Andrey Zolotarev, Artem Selyutin, Sergey Ermakov, Anastasia Penkova

**Affiliations:** St. Petersburg State University, 7/9 Universitetskaya nab., 199034 St. Petersburg, Russia

**Keywords:** cellulose derivatives, cellulose acetate, carboxymethyl cellulose, Zn-based metal–organic frameworks, nanofiltration, water treatment, heavy metals

## Abstract

Nowadays, nanofiltration is actively used for water softening and disinfection, pre-treatment, nitrate, and color removal, in particular, for heavy metal ions removal from wastewater. In this regard, new, effective materials are required. In the present work, novel sustainable porous membranes from cellulose acetate (CA) and supported membranes consisting of CA porous substrate with a thin dense selective layer from carboxymethyl cellulose (CMC) modified with first-time synthesized Zn-based metal–organic frameworks (Zn(SEB), Zn(BDC)Si, Zn(BIM)) were developed to increase the efficiency of nanofiltration for the removal of heavy metal ions. Zn-based MOFs were characterized by sorption measurements, X-ray diffraction (XRD), and scanning electron microscopy (SEM). The obtained membranes were studied by the spectroscopic (FTIR), standard porosimetry and microscopic (SEM and AFM) methods, and contact angle measurement. The CA porous support was compared with other, prepared in the present work, porous substrates from poly(m-phenylene isophthalamide) and polyacrylonitrile. Membrane performance was tested in the nanofiltration of the model and real mixtures containing heavy metal ions. The improvement of the transport properties of the developed membranes was achieved through Zn-based MOF modification due to their porous structure, hydrophilic properties, and different particle shapes.

## 1. Introduction

Wastewater from various industrial sources is a huge threat to the ecological balance and human health due to its toxicity and pollution [1]. In particular, the increase in industrial water pollution with heavy metal ions is of global concern. Industries, such as metallurgy, battery production, electroplating, metal finishing, etc., are the main sources of heavy metal ions [2]. Traditional methods (photocatalysis, electrodialysis, precipitation, degradation, ion exchange, etc.) widely used for wastewater treatment for the removal of heavy metal ions [3] have such disadvantages as low separation efficiency and the application of additional chemicals resulting in pollution [4]. Most of the recent research for water treatment from heavy metal ions has focused on adsorption methods. Different types of adsorbents were developed for wastewater remediation: carbon-based [5,6]; chitosan-based [7]; mineral; magnetic, metal–organic framework adsorbents; and biosorbents [8]. The main obstacles to adsorption methods are the possibility of simultaneous removal of various types of ions, resistance to adsorbent cycling, and long retention times [8]. In recent years, technological advances in membrane development have led to an increase in the use of membranes for the extraction of heavy metal ions from wastewater. Nanofiltration (NF) is a pressure-driven membrane method considered an environmentally friendly and energy-efficient separation process for water treatment and one of the most advanced separation technologies for removing heavy metal ions from water [9]. 

Currently, various strategies for the development of efficient and inexpensive membranes based on biopolymers (carboxymethyl cellulose, chitosan, sodium alginate, and cellulose acetate) for wastewater treatment are being actively studied [10]. Biopolymers as membrane materials are of great interest due to increased hydrophilic permeability and excellent selectivity toward heavy metal ions, and they can be destroyed by living organisms during disposal [11,12]. In membrane technology, biopolymer membranes are still an expanding market. Cellulose is one of the most important natural polymers on the market, but it is difficult to process due to its low solubility in most solvents. To solve this problem, various cellulose derivatives are produced by several methods and are widely used in industry to reduce the content of microorganisms in raw water in reverse osmosis and ultrafiltration processes [13,14].

One of the first commercially successful polymeric membranes with high performance and high salt retention was an asymmetric CA membrane manufactured in the early 1960s [15]. Nowadays, active research is also ongoing for the use of CA as a matrix for nanofiltration membranes. Porous CA membranes are being actively developed through the modification with ZnO and TiO_2_ nanoparticles [16] and Zn-based metal–organic framework (MOF-5) [2], application of glycerol derivatives, methyl lactate as solvents and 2-methyltetrahydrofuran (2-MeTHF) as co-solvent [17,18], and variation of the casting solution concentration [19] for nanofiltration of dithioterethiol (DTT), aqueous solutions of Cu (II) and Co (II), rose bengal (RB), MgSO_4_, and Cd^2+^ ions, respectively. In this work [10], nanofiltration dense CA/vinyl-triethoxysilane-modified graphene oxide (GO) and gum Arabic (GuA) membranes were investigated for the removal of Pb (II).

Recently, nanofiltration membranes have been actively developed from carboxymethyl cellulose (CMC), which has good hydrophilicity and a combination of cross-linkable hydroxyl and carboxylic acid groups [20]. The development of nanofiltration cross-linked with GA-supported CMC membranes on the polyethersulfone (PES) [21] and polysulfone (PS) substrates [20] was carried out for the separation of aqueous solutions with single-salt composition (of Na^+^, K^+^, and Mg^2+^) and dyes (the xylenol orange (XO) and methyl blue (MYB)) [20]. In the works [22,23], CMC was blended with polyvinyl alcohol (PVA) to develop supported membranes for nanofiltration of different inorganic electrolytes (NaCl, Na_2_SO_4,_ MgSO_4_, MgCl_2_) and dye (methyl blue and congo red) solutions. Nanofiltration hollow fiber membranes, consisting of one polyelectrolyte bilayer of CMC and polyethylenimine (PEI) [24], CMC cross-linked with AlCl_3_ [25] and with FeCl_3_ [26] on polypropylene (PP) substrate, were developed for separation of aqueous solutions, containing MgCl_2_, CaCl_2_, KCl, NaCl, Na_2_SO_4_, MgSO_4_, and polyethyleneglycol (PEG) of different molecular weights and organic dyes. Polyelectrolyte complexes (PEC) of CMC with poly(2-methacryloyloxy ethyl trimethylammonium chloride) (PDMC) [27] and quaternary ammonium cellulose ether (QCMC) [28] were synthesized for the development of nanofiltration supported membranes for the separation of K_2_SO_4_ and xylenol orange solutions. 

Over the years, many efforts have been made in the field of nanofiltration cellulose derivative-based membranes to improve the separation process and membrane stability. However, based on the literature review, there are no studies of CA and CMC-based membranes in the nanofiltration of solutions of a heavy metal ions mixture for water treatment, in particular, their testing for real objects—wastewater from the industry. Additionally, the use of these polymeric membranes in nanofiltration was limited by the insufficiency of their mechanical, chemical resistance, and transport characteristics (low permeability and/or rejection coefficients) for their promising use in industry. Thus, in this work to improve the porous CA and supported CMC/CA membranes performance in nanofiltration of solutions of heavy metal ions mixture, the modification with first synthesized porous Zn-based metal–organic frameworks (MOFs)—Zn(SEB), Zn(BDC)Si, and Zn(BIM), which have proven themselves as good fillers of polymeric membranes for nanofiltration [9], was carried out. It is also worth noting that there are no reported literature data on the modification of any polymer with novel Zn(SEB), Zn(BDC)Si particles, and Zn(BIM) with the atypical structure for Zn-based MOFs for nanofiltration.

The aim of this work was to develop novel, highly-efficient sustainable porous membranes from cellulose acetate (CA) and supported membranes consisting of CA porous substrate with a thin dense selective layer from carboxymethyl cellulose (CMC) modified with first-time synthesized Zn-based metal–organic frameworks (Zn(SEB), Zn(BDC)Si, Zn(BIM)) for enhanced nanofiltration for the removal of heavy metal ions. To evaluate the properties of the CA porous substrate, the supported CMC/CA membranes were compared with other, prepared in the present work, porous substrates from poly(m-phenylene isophthalamide) and polyacrylonitrile. The improvement of the transport properties of the developed membranes was achieved through Zn-based MOF modification due to their porous structure, hydrophilic properties, and different particle shapes. Zn-based MOFs were characterized by sorption measurements, X-ray diffraction (XRD), and scanning electron microscopy (SEM). The resulting membranes were studied by spectroscopic (FTIR), standard porosimetry, microscopic (SEM and AFM) methods, and contact angle measurement. Transport properties of the membranes were evaluated in nanofiltration of the model and real mixtures containing heavy metal ions.

## 2. Materials and Methods

### 2.1. Materials

Cellulose acetate (CA, Mn = 40,000 g/mol, CDA-F, Alfa Laval, Copenhagen, Denmark) was used as a matrix for the preparation of porous membranes. Carboxymethyl cellulose (CMC, Mn = 400,000 g/mol, Bioprod LLC, St. Petersburg, Russia) was used for the formation of a dense thin selective layer of supported membranes. Zn-based metal–organic frameworks (MOFs) as Zn(SEB) (sebacic acid as a ligand), Zn(BDC)Si (2-trimethylsilylterephthalic acid (TSTA) as a ligand), Zn(BIM) (benzimidazole as a ligand) synthesized at the Saint-Petersburg State University (St. Petersburg, Russia) were used for the modification of the developed nanofiltration membranes (the synthesis and characterization of Zn-based MOFs are presented in Section S1 of Supplementary Materials). Poly(m-phenylene isophtalamide) (PA, Fenylon C2, lot. 146/19, “UNIPLAST” Ltd., Vladimir, Russia) and polyacrylonitrile (PAN, COA No.: A05P10833, Mw = 150,000 g/mol, Ming International Co., St. Petersburg, Russia) were used as porous substrates for the development supported CMC membranes for the comparison. N,N′-dimethylacetamide (DMAc), glutaraldehyde (GA), and sulfuric acid (H_2_SO_4_) purchased from “Vekton” (St. Petersburg, Russia) were used without additional treatment.

### 2.2. Membrane Preparation

The modification with Zn-based MOF (Zn(SEB), Zn(BDC)Si, and Zn(BIM)) of CA or CMC matrices was carried out by the solid-phase method grinding the calculated amount of polymers with Zn-based MOF powders and obtaining a dispersion in a polymer solvent [9,29].

#### 2.2.1. Porous Membranes

CA solutions were prepared as follows: the pre-determined amount of CA powder was dissolved in DMAc) to obtain casting solutions with concentrations of 12, 15, 17, and 20 wt.% at ambient temperature under constant stirring and the following sonication. Porous CA membranes were formed by the phase inversion method via a non-solvent induced phase separation (NIPS) technique [29,30]: the polymer solution was cast by a casting blade with a gap width of 200 μm onto a glass support, which then was immersed in a coagulation bath with methanol at 25 °C. After the formation, all prepared membranes were left in a water bath for 12 h at ambient temperature to remove the remaining solvents from the structure of membranes [16]. The modified porous membranes were prepared according to the technique for the unmodified CA membranes with the introduction of 0.5–1.5 wt.% Zn(SEB), 1 wt.% Zn(BDC)Si, and Zn(BIM) with respect to the polymer weight.

#### 2.2.2. Supported Membranes

The supported CMC membrane was prepared by physical adsorption as follows [31]: 1 wt.% CMC solution dissolved in distilled water at 40 °C under constant stirring, and the following sonication was deposited onto porous membranes (substrates) from CA, Poly(m-phenylene isophtalamide) (PA), and polyacrylonitrile (PAN) with the following solvent evaporation at ambient temperature for 12 h to form a thin dense selective layer. Porous PA and PAN substrates were prepared without the use of a polyester substrate from 15 wt.% casting polymer solutions by the phase inversion method via a non-solvent induced phase separation (NIPS) technique according to the previously described in the work’s procedures [32,33]. To use supported membranes in nanofiltration of aqueous heavy metal ions solutions, the cross-linking of CMC chains was carried out by the immersion of the supported membranes in an aqueous solution containing 1 wt.% glutaraldehyde (GA) and 0.5 wt.% sulfuric acid (H_2_SO_4_) for 1 min. After this, membranes were dried in the air for 30 min and in the oven at 60 °C for 10 min with the following washing with distilled water [23]. The modified supported membranes were prepared according to the technique for the unmodified CMC membranes with the introduction of 5–15 wt.% Zn(SEB), Zn(BDC)Si or Zn(BIM) with respect to the polymer weight.

### 2.3. Nanofiltration Experiment

Transport properties of the developed porous CA and supported CMC membranes were tested in nanofiltration of model solutions of heavy metal ions (Cu(NO_3_)_2_, Pb(NO_3_)_2_, and Cd(NO_3_)_2_, 50 mg/L of each salt) and a real object—wastewater from galvanic production (LLC “Galvanik”, St. Petersburg, Russia) using a laboratory dead-end cell (effective area of 0.2·10^−2^ m^2^) with constant stirring at 22 °C [9,34]. The scheme of the nanofiltration was presented in the previous work [9]. Nanofiltration experiments were carried out for at least a week for each membrane, and the data presented were averaged.

The content in the feed and permeate of metal ions of model solutions was investigated using a TA-4 voltammetric analyzer by stripping voltammetry. The silver chloride electrodes were used as a reference, auxiliary electrodes, and a mercury film electrode as a working electrode. The content in the feed and permeate of metal ions (Cd^2+^, Cr^3+^, Cu^2+^, Ni^2+^, Zn^2+^) of the wastewater from galvanic production was analyzed using an ICPE-9000 optical emission spectrometer (Shimadzu, Japan) by atomic emission spectrometry. The calibration solutions with elements (Cd^2+^, Cr^3+^, Cu^2+^, Ni^2+^, Zn^2+^) were prepared in 0.1 N HNO_3_ with the MERCK multielement standard. Wavelengths for metal ions Cd^2+^, Cr^3+^, Cu^2+^, Ni^2+^, and Zn^2+^ were as follows: 228.802 nm, 205.552 nm, 327.396 nm, 231.604 nm, and 206.200 nm [9].

Permeability (*L*, kg/(m^2^∙h∙atm)) was calculated as the ratio of permeation flux to the transmembrane pressure (*∆P*) according to Equation (1):

(1) $$L=\frac{J}{\Delta P}=\frac{m}{A\cdot t\cdot \Delta P},$$

where *m* is the permeate weight (kg), *A* is effective membrane surface area (m^2^), *t* is a time of the permeate collection (h), and Δ*P* is nanofiltration pressure (atm).

The rejection coefficient (*R*, %) of the heavy metal ions was calculated according to Equation (2):

(2) $$R=(1-\frac{C_{perm}}{C_{feed}})\cdot 100\%,$$

where *C_perm_* and *C_feed_* are the heavy metal ions concentration in the permeate and the feed, respectively.

### 2.4. Fourier-Transform Infrared Spectroscopy

The structural changes of the developed CA and CMC-based membranes were investigated by Fourier-transform infrared spectroscopy (FTIR) using an IRAffinity-1S spectrometer (Shimadzu, St. Petersburg, Russia) and an attenuated total reflectance accessory (PIKE Technologies, St. Petersburg, Russia) in the range of 400–4000 cm^−1^ at 25 °C.

### 2.5. Scanning Electron Microscopy

The surface and cross-sectional morphology of the developed membranes was studied by scanning electron microscopy (SEM) using a Zeiss Merlin SEM (Carl Zeiss SMT, Oberhochen, Germany) at an accelerating voltage of 1 kV and electron beam current of 100 pA to prevent surface charging.

### 2.6. Atomic Force Microscopy

NT-MDT NTegra Maximus atomic force microscope (NT-MDT Spectrum Instruments, Moscow, Russia) in the tapping mode with standard silicone cantilevers (hardness of 15 N/m) was used to study the surface topography of the developed CA and CMC-based membranes by atomic force microscopy (AFM).

### 2.7. Contact Angle Measurement

To assess the effect of modification on the surface hydrophilic–hydrophobic balance of membranes, the contact angles were measured by the sessile drop (for supported CMC-based membranes) [35] and attached bubble method (for porous CA-based membranes) [36] using a Goniometer LK-1 (OOO NPK Open Science, Krasnogorsk, Russia). The water contact angles for supported membranes were evaluated only on the side of the dense CMC-based selective layer. The DropShape software was used to analyze the data obtained. For each membrane, the measurements were obtained at least in three different positions, and average values of contact angle were calculated and presented.

### 2.8. Standard Porosimetry Method

The total porosity of the porous membranes was investigated using a Porosimeter 3.1 instrument (Porotech Ltd., Ottawa, ON, Canada) with n-octane as the reference liquid at 30 °C by the standard porosimetry method [37].

### 2.9. Mechanical Properties Investigation

Membrane mechanical properties (maximum tension and elongation) were studied using the Shimadzu AG-50kNXD autograph (Shimadzu, Japan) and ASTM D638, ISO 527–2 protocols.

## 3. Results

This section has three main parts. Section 3.1 is devoted to the development and investigation of porous CA-based membranes. Section 3.2 concerns the development and investigation of supported membranes with a thin dense CMC-based layer deposited onto the developed porous CA-based membrane (applied as a substrate). Section 3.3 is dedicated to the study of the developed membranes in nanofiltration of a real object—wastewater from galvanic production to demonstrate the perspective application for industrial purposes.

### 3.1. Development and Investigation of Porous CA-Based Membranes

#### 3.1.1. Transport Properties of the Porous CA-Based Membranes

**Figure 1:**
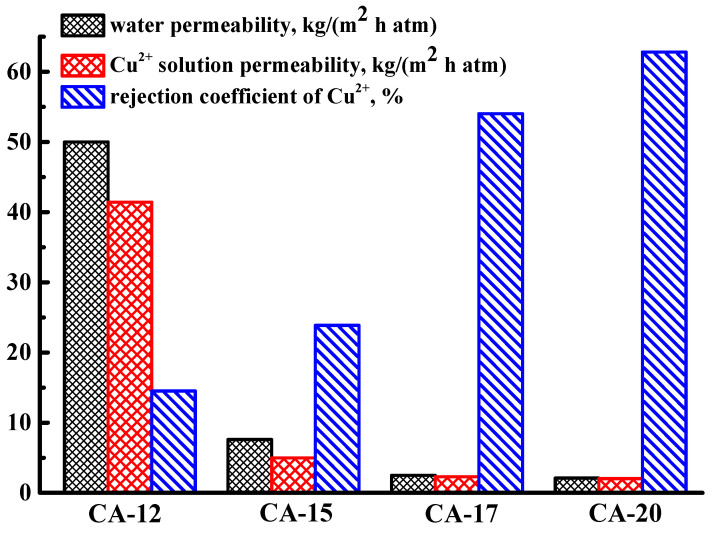
Transport properties of CA-based membranes prepared from different casting solution concentrations in nanofiltration of water and a model Cu^2+^ solution.

To study the effect of the polymer solution concentration, CA-based membranes were prepared from casting solutions with concentrations of 12 wt.% (CA-12), 15 wt.% (CA-15), 17 wt.% (CA-17), and 20 wt.% (CA-20) were tested in the nanofiltration of water and model Cu^2+^ solution (Figure 1).

**Figure 2:**
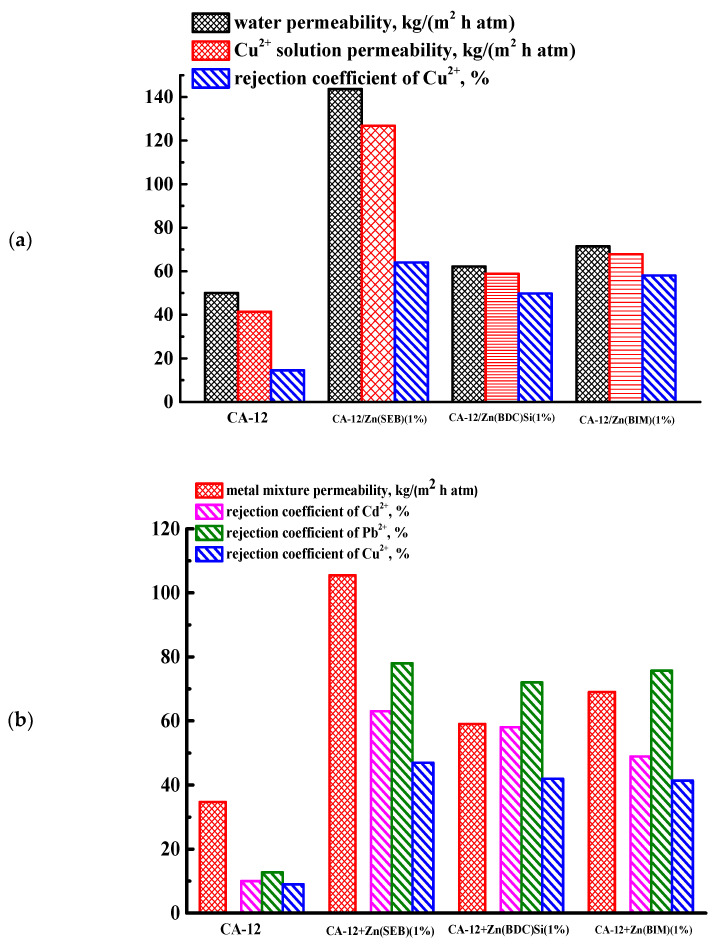
Transport properties of CA-12 and CA-12/Zn-based MOFs (Zn(SEB), Zn(BDC)Si, Zn(BIM)) membranes in nanofiltration of (**a**) water and model Cu^2+^ solution, and (**b**) a model solution of heavy metal ions (Cd^2+^, Pb^2+^, Cu^2+^).

It was found that an increase in the concentration of CA solution led to a decrease in the permeability of both water and Cu^2+^ solution and an increase in the rejection coefficient (R) of copper. It was due to the decrease in the pore size (especially macrovoids) and an increase in the thickness of the dense top layer of the CA-based membranes with the rise of CA concentration in the casting solution that resulted in the higher density of the polymer solution (confirmed by SEM data in Figure 5 presented below). The highest permeability values (50 kg/(m^2^h atm) for water and 41 kg/(m^2^h atm) for Cu^2+^ solution) with a 15% rejection coefficient of copper were noted for the CA-12 membrane (prepared from 12 wt.% castings CA solution), which was chosen for further modification with Zn-based MOFs in order to improve the permeability and selectivity. A total of 1 wt.% Zn(SEB), Zn(BDC)Si, and Zn(BIM) were introduced into the CA-12 membrane matrix to select the optimal modifier and to study the effect of the MOF modification on the nanofiltration performance. The modified Zn-based MOF membranes were tested in nanofiltration of water, model Cu^2+^ solution, and solution of heavy metal ions mixture containing Cd^2+^, Pb^2+^, and Cu^2+^ ions (Figure 2). The transport parameters for the CA-12 membrane in nanofiltration of water and model Cu^2+^ solution were presented in Figure 2a for comparison with the modified membranes.

It was found that the introduction of 1 wt.% Zn-based MOFs into the CA matrix led to an increase in the permeability of both water and heavy metal ions solutions and rejection coefficient values. The increased permeability of the modified membranes was because of the combined effects: the high affinity of Zn-based MOFs toward water due to their hydrophilic nature [38], increased inner porosity attributed to the changes in the number and size of macrovoids (confirmed by SEM data in Figure 6 presented below) and surface hydrophilicity (confirmed by contact angle data in Table 1 presented below) [39]. Zn-based MOFs may form hydrogen bonds with water, thereby developing novel transport channels for its molecules [39,40,41]. The intrinsic size of MOF pores and the formation of novel transport channels in the CA matrix with an increased surface hydrophilicity facilitated water transfer through the membranes, increasing permeability. An increase in the selectivity of the CA-12/Zn-based MOF membranes could be due to the structure of the membranes’ top dense layer, which became denser and thicker during modification compared to the pristine CA-12 membrane (confirmed by SEM data below) [39], as well as the presence of MOFs with the pore structure (considered in Section S1 of Supplementary Materials), which positively affect the metal ions rejection coefficients. The introduction of Zn(SEB) and Zn(BIM) into the CA-12 matrix contributed to higher permeability compared to the CA-12/Zn(BDC)Si (1%) membrane. It may be explained by the higher affinity of these Zn-based MOFs toward water due to their more hydrophilic nature compared to Zn(BDC)Si. Zn(BDC)Si has the most hydrophobic nature among all MOF particles due to the trimethylsilyl groups inside the pores. Earlier in the work [42], it was also demonstrated that SIFSIX-3-Ni MOF, despite a low surface area, had more saturation with CO_2_ compared to the MOF with a higher specific surface area due to a very high affinity toward CO_2_. Furthermore, for all CA-based membranes, the copper rejection coefficient in nanofiltration of a model solution of heavy metal ions (Cd^2+^, Pb^2+^, Cu^2+^) significantly decreased compared to nanofiltration of a Cu^2+^ solution. It was associated with the copper formation of labile complexes with donor atoms and different dynamic sorption characteristics of metal ions [9,34]. The highest transport parameters were noted by the CA-12/Zn(SEB) (1%) membrane: water permeability of 144 kg/(m^2^h atm), Cu^2+^ solution permeability of 127 kg/(m^2^h atm) and permeability of solution of heavy metal ions—105 kg/(m^2^h atm) with rejection coefficients for Cd^2+^, Pb^2+^, Cu^2+^—63, 78, and 47%, respectively. The improved permeability may be due to better dispersion of this Zn-based MOF in the CA matrix, an increase in the macrovoid size in the inner porous membrane structure (confirmed by SEM data in Figure 6) and surface hydrophilization (confirmed by contact angle data in Table 1) of the modified CA-12/Zn(SEB) (1%) membrane because of the peculiarity of shape (the smallest particle size), the biggest pore size and porosity of the Zn(SEB) particles (confirmed by physisorption measurements, X-ray diffraction (XRD) analysis, and SEM data in Figures S2–S5 of Supplementary Materials). The increase in rejection coefficients for CA-12/Zn(SEB) (1%) membrane was attributed to the pore structure of this MOF, where hydrophobic hydrocarbon tails stick out inside the pores, limiting the transfer of metal ions (confirmed by the structure of this MOF in Figure S4 of Supplementary Materials). Thus, Zn(SEB) was chosen as the optimal modifier for the CA-12 membrane due to the highest productivity (permeability and rejection coefficients) of the membrane modified by it.

**Figure 3:**
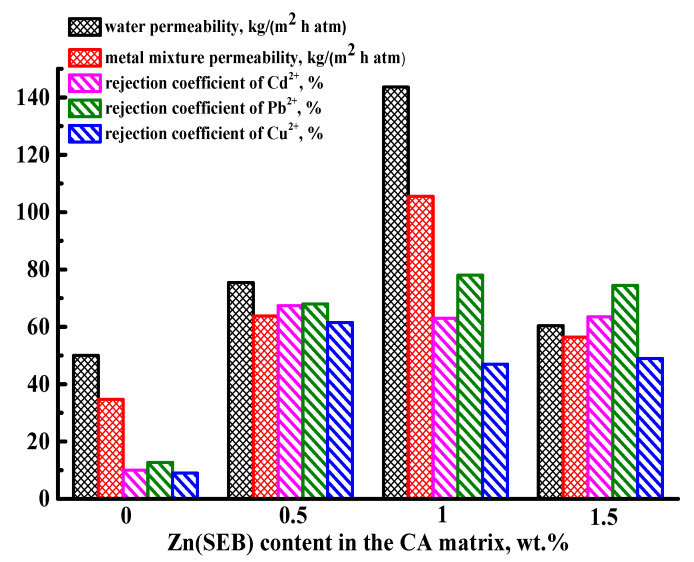
Transport properties of CA-12 and CA-12/Zn(SEB) (0.5, 1, and 1.5 wt.%) membranes in nanofiltration of water and a model solution of heavy metal ions (Cd^2+^, Pb^2+^, Cu^2+^).

To select the optimal concentration of this modifier, the CA-12 membrane was modified with various concentrations of Zn(SEB) (0.5, 1, and 1.5 wt.% with respect to the CA weight). Transport properties of these CA-12/Zn(SEB) membranes were evaluated in nanofiltration of water and a model solution of heavy metal ions (Cd^2+^, Pb^2+^, Cu^2+^) (Figure 3). The parameters for the CA-12/Zn(SEB) (1%) membrane were represented in Figure 3 for comparison.

An increase to 1 wt.% in the Zn(SEB) content in the CA matrix led to an increase in the permeability of water and model solution of heavy metal ions. The introduction of 1.5 wt.% Zn(SEB) already led to a decrease in this parameter. This could be due to the possible agglomeration of MOF particles because of the higher content in the membrane, which hindered the mass transfer of components through the membrane reducing its permeability [43,44]. The rejection coefficient values for CA-12/Zn(SEB) membranes with 1 and 1.5% MOF were relatively the same for all metal ions, while for the CA-12/Zn(SEB) (0.5%) membrane, they were slightly higher for Cd^2+^ and Cu^2+^. This may be due to the fact that the higher Zn(SEB) content in the CA-12 membrane can lead to the blockage of some small membrane pores [45], slightly reducing retention. Thus, the CA-12/Zn(SEB) (1 wt.%) membrane possessed the highest permeability of a model solution of heavy metal ions (two times compared to the CA-12 membrane) and increased rejection coefficients for Cd^2+^, Pb^2+^, Cu^2+^ metal ions (53, 65 and 38% more compared to the CA-12 membrane). It was chosen for testing in nanofiltration of water and a model solution of heavy metal ions (Cd^2+^, Pb^2+^, Cu^2+^) for six days (Figure S7a of Supplementary Materials) to confirm its stability and reusability and in nanofiltration of real wastewater from galvanic production (presented in Section 3.3).

#### 3.1.2. Characterization of the Porous CA-Based Membranes

**Figure 4:**
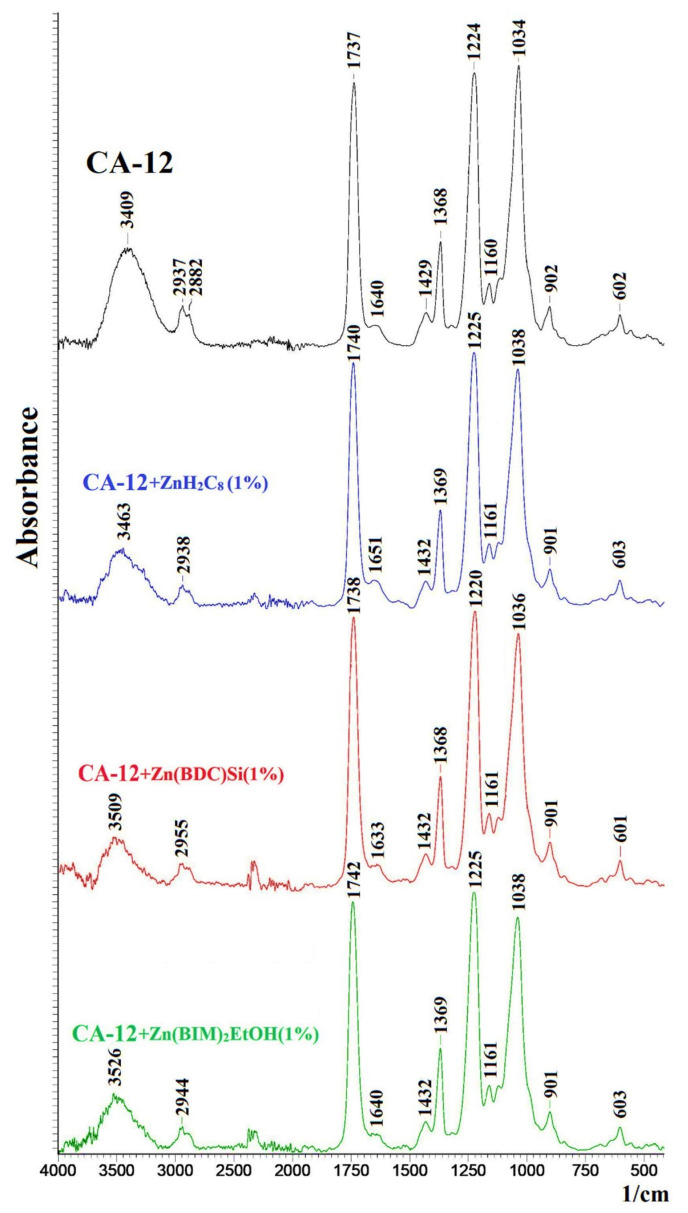
FTIR spectra of nanofiltration porous CA-12 and CA-12/Zn-based MOF membranes.

To explain the obtained dependences of the transport characteristics of porous CA-based membranes, their structure, and physicochemical properties were studied by FTIR spectroscopy, SEM and AFM microscopies, contact angle, and porosity measurements. FTIR spectra of the nanofiltration porous CA and CA/Zn-based MOF membranes are presented in Figure 4.

The FTIR spectrum of the CA-12 membrane had characteristic peaks at 3409, 1737, 1224, and 1034 cm^−1^, related to vibrations of hydroxyl groups, stretching of the C-O group, aromatic ring, and of the –C-O- bond in the CH_2_-OH group, respectively [10,16,46]. The introduction of Zn-based MOFs practically did not change the FTIR spectra of the modified membranes due to a low modifier content [47] as the characteristics peak of hydroxyl groups at 3409 cm^−1^ was shifted with significant decreased intensity [16], and peaks at 2937, 1737, 1224, and 1034 cm^−1^ had only slight shifts. These changes may indicate the formation of hydrogen bonds or complexes between MOFs and CA [16,48]. The XRD profiles of modified CA-based membranes showed the absence of characteristic peaks of Zn-based MOFs due to a small amount (1 wt.%) of them in the membrane matrix (Figure S6 of Supplementary Materials).

**Figure 5:**
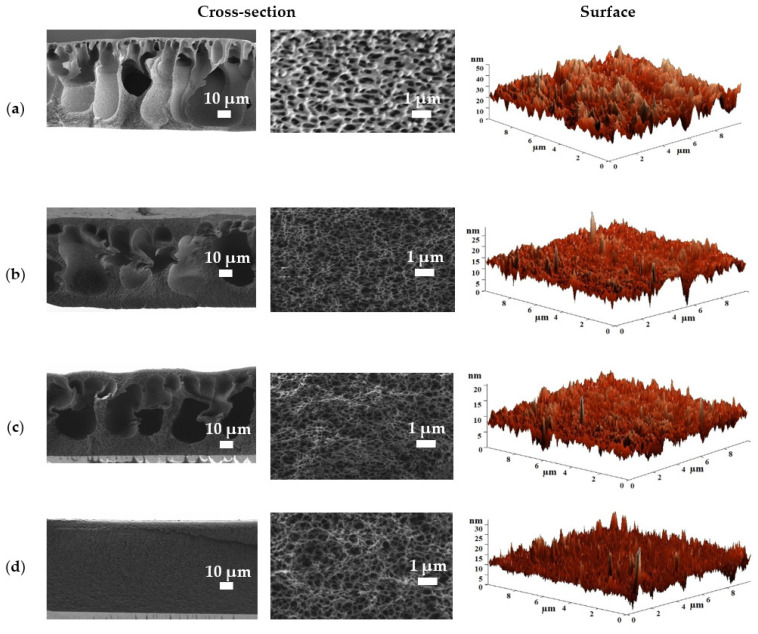
Cross-sectional SEM micrographs at different magnifications and surface AFM images of (**a**) CA-12, (**b**) CA-15, (**c**) CA-17, and (**d**) CA-20 membranes.

**Figure 6:**
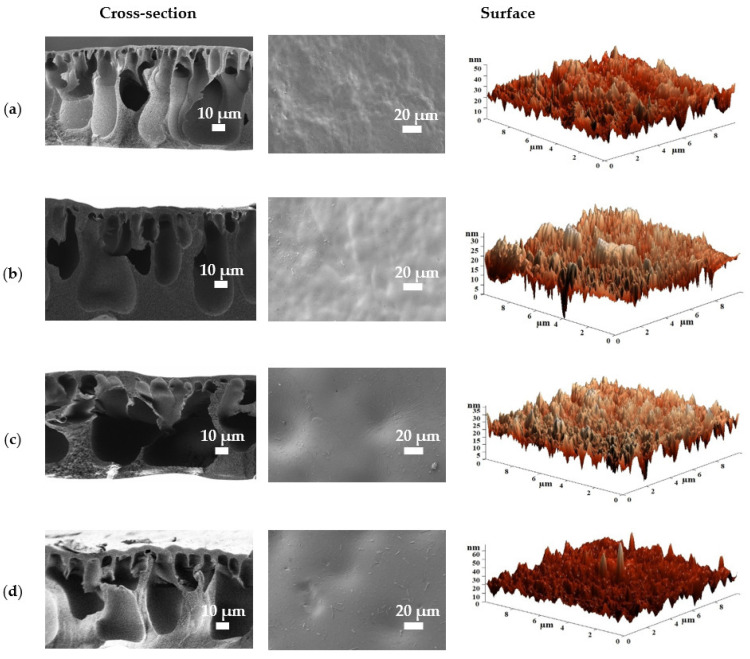
Cross-sectional and surface SEM micrographs and surface AFM images of (**a**) CA-12, (**b**) CA-12/Zn(SEB) (1%), (**c**) CA-12/Zn(BDC)Si (1%), and (**d**) CA-12/Zn(BIM) (1%) membranes.

The surface and cross-sectional morphology and surface topography of the porous CA-based membranes were studied by SEM and AFM (Figure 5 and Figure 6).

Cross-sectional SEM micrographs showed that the pore size of the CA-based membranes decreased with an increase in the CA concentration in the casting solution from 12 to 20 wt.% (Figure 5), and the CA-20 membrane, prepared from the casting solution with the highest CA concentration of 20 wt.%, was characterized by the absence of large macrovoids (“finger-shaped” pores) in the cross-sectional structure (Figure 5d). The higher polymer concentration in the casting solution led to the membrane formation with a lower porosity [16,19]. The CA-12 membrane has a finger-shaped pore structure (macrovoids) with a thin dense upper layer, which indicates instantaneous demixing during the NIPS process; the polymer precipitates quickly after immersion in a coagulation bath [49], while for the CA-15 and CA-17 membranes, the substructure changes slightly (in the form of vacuole-shaped pore structure) and the dense upper layer thickens with increasing polymer concentration in the casting solution. The membrane prepared from 20 wt.% casting solution (CA-20) has a spongy substructure and a relatively thick dense top layer, indicating delayed demixing (the precipitation takes a longer time) due to the high density of the polymer solution. When the polymer content is above a certain threshold, there is not a sufficient exchange of non-solvent and solvent to form large pores (macrovoids) during phase separation and solidification [49], so the porosity of the membrane decreases (confirmed by total porosity data below), causing a decrease in permeability (Figure 1).

The introduction of 1 wt.% Zn-based MOFs (Zn(SEB), Zn(BDC)Si and Zn(BIM)) leads to morphology changes in both cross-sectional and surface structure of the CA-12 membranes (Figure 6), affecting the nanofiltration characteristics (Figure 2). The modified membranes had a denser and thicker top layer in the cross-section compared to the pristine CA-12 membrane, resulting in higher metal ions rejection. The modification of the CA-12 membrane with Zn(SEB) led to an increase in macrovoid sizes in the cross-sectional structure maintaining a similar form of the membrane surface compared to the unmodified membrane. The Zn(SEB) particles were well-dispersed in the CA matrix as there was no agglomeration of the filler on the membrane surface due to the smallest size of particles (confirmed by SEM data in Figure S5 of Supplementary Materials) [50]. Furthermore, recent studies have shown that MOFs with smaller particle sizes are more compatible with polymers [51,52]. The CA-12/Zn(BDC)Si (1%) membrane had the largest macrovoid size of the cross-section due to the effect of the introduction of the Zn(BDC)Si with the biggest particle size (confirmed by SEM data in Figure S5 of Supplementary Materials). The membrane with Zn(BIM) showed a change in macrovoid shape from a finger-like to a vacuole-like one compared to the CA-12 membrane. This may be due to the needle-shaped Zn(BIM) structure (confirmed by SEM data in Figure S5 of Supplementary Materials), which is atypical for the Zn-based MOFs. It is also worth noting that the Zn-based MOF particles on the surface were observed to a greater extent for CA-12/Zn(BDC)Si (1%) and CA-12/Zn(BIM) (1%) membranes, which could indicate a lesser dispersion of these modifiers in the CA matrix due to the peculiarity of their shapes (confirmed by SEM data in Figure S5 of Supplementary Materials) [53].

Based on the AFM images shown in Figure 5 and Figure 6, the average surface roughness (Ra) of the CA-based membranes was calculated (Table 1). To assess the hydrophilic–hydrophobic balance of the membrane surface, the contact angles were measured, and the total porosity of the membranes was also evaluated. The data obtained are presented in Table 1.

It was found that the increase in CA concentration (from 12 to 20 wt.%) in the casting solution led to a slight decrease in the average surface roughness (from 3.16 to 1.60 nm) and in membrane total porosity (from 95.8 to 91.8%) [54]. This was due to an increase in the casting solution viscosity, which led to the formation of a denser porous structure of the CA membranes, also confirmed by SEM micrographs (Figure 5) [49]. It is also worth noting a slight increase in the contact angle values of the CA membranes with an increase in the casting solution concentration from 12 to 20 wt.%. This effect has also been observed for the porous polysulfone membranes in work [55].

The introduction of 1 wt.% Zn-based MOFs into the CA-12 membrane did not significantly change the average surface roughness and resulted in a decrease in the contact angle, the total porosity, and porosity over the weight of porous nanofiltration membranes compared to the unmodified CA-12 membrane (Table 1). The hydrophilization of the membrane surface was attributed to the hydrophilic Zn-MOF nature [38]. The decrease in the total porosity and porosity over weight values of the modified membranes was caused by the morphology changes during the modification (confirmed by SEM data, Figure 6) and could be due to the possible blockage of small pores by MOF particles [45]. The CA-12/Zn(SEB) (1%) membrane had the lowest Ra, contact angle, and the highest total porosity values among CA-12/Zn-based MOF membranes due to Zn(SEB) structure features (the smallest particle size and the highest porosity, confirmed by physisorption measurements and SEM data in Figures S2 and S5 of Supplementary Materials) and better dispersion in the polymer matrix. The mechanical properties of the porous CA-12 and CA-12/Zn(SEB) (1%) membranes were also compared. It was demonstrated that the introduction of 1 wt.% Zn(SEB) into the CA matrix led to the improvement of mechanical properties: from 0.13 to 0.5 MPa for maximum tension and from 1.83 to 1.97 mm for maximum elongation. It should be noted that composite membranes generally exhibit improved mechanical properties [56]. All these changes are reflected in the transport properties of the modified membranes in nanofiltration (Figure 2).

### 3.2. Development and Investigation of Supported CMC-Based Membranes

The nanofiltration-supported CMC-based membranes were prepared by the deposition of a thin dense CMC and CMC/Zn-based MOF layer onto the porous CA-based membrane (applied as a substrate) developed in Section 3.1. Additionally, to use CMC-based membranes for nanofiltration of aqueous solutions, cross-linking of polymer chains with glutaraldehyde (GA) was carried out. The creation of such a type of supported membranes allowed increasing membrane rejection of heavy metal ions for water treatment by nanofiltration due to the dense selective polymer layer.

#### 3.2.1. Transport Properties of the Supported CMC-Based Membranes

**Figure 7:**
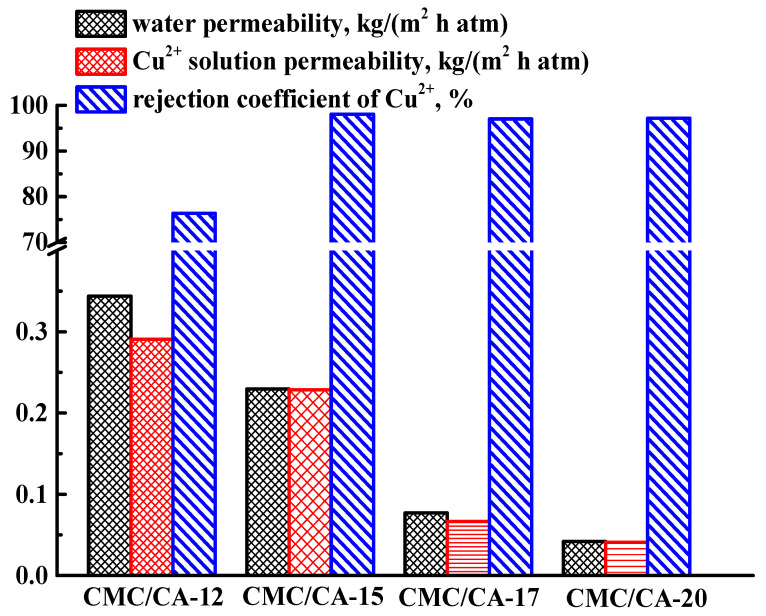
Transport properties of the supported CMC/CA membranes in nanofiltration of water and model Cu^2+^ solution.

To study the effect of a porous structure of the CA-based membrane (further as a substrate) on the transport properties, a thin selective layer of the CMC was deposited on the porous CA-based membranes (CA-12, CA-15, CA-17, and CA-20), prepared from the various concentration of casting solution. The obtained supported CMC/CA membranes were tested in the nanofiltration of water and a model Cu^2+^ solution (Figure 7).

It was demonstrated that the porous structure of the CA-based substrate affected the permeability and rejection of the supported membranes. The permeability values of the supported CMC/CA membranes decreased with an increase in the concentration of the casting solution (12–20 wt.% of CA) from which the CA-based was prepared. It was due to the decrease in the pore size (especially, macrovoids) and total porosity of the CA-based substrate (confirmed by SEM and total porosity data in Figure 5 and Table 1, respectively). For the CMC/CA-12 membrane, the highest permeability (0.34 kg/(m^2^ h atm) for water and 0.29 kg/(m^2^ h atm) for copper solution) and the lowest rejection coefficient of Cu^2+^ (76%) were observed, which could be due to the penetration of the CMC polymer into the pores of the substrate with the highest porosity. While for other membranes (CMC/CA-15, CMC/CA-17, and CMC/CA-20), the rejection coefficient of Cu^2+^ values was relatively the same (97–98%), indicating a uniform and defect-free thin selective layer based on CMC, which ensured high selectivity to heavy metal ions. Thus, the optimal substrate for the CMC was a porous membrane based on CA prepared from a 15 wt.% casting solution, as the CMC/CA-15 membrane had the increased permeability (0.23 kg/(m^2^ h atm) for water and copper solution) compared to the CMC/CA-17 and CMC/CA-20 membranes with a high level of the copper rejection (98%). 

**Figure 8:**
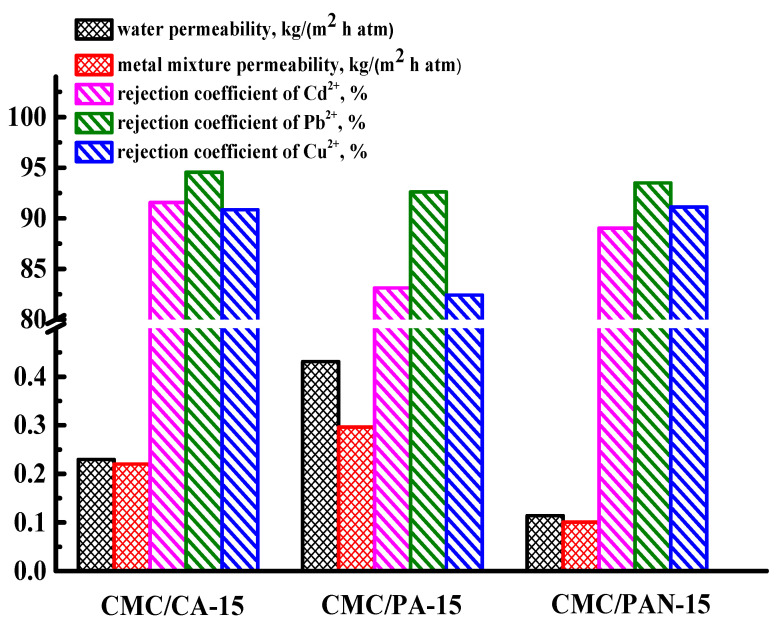
Transport properties of the supported CMC-based membranes in nanofiltration of water and a model solution of heavy metal ions (Cd^2+^, Pb^2+^, Cu^2+^).

For comparison, other cross-linked supported CMC-based membranes were prepared on the developed porous substrates based on poly(m-phenylene isophtalamide) (PA-15) and polyacrylonitrile (PAN-15). The transport properties of the prepared supported CMC-based membranes on the various substrates were evaluated in nanofiltration of water and a model solution of heavy metal ions (Cd^2+^, Pb^2+^, Cu^2+^) (Figure 8).

The CMC/PA-15 membrane was the most permeable, with a low level of heavy metal ion rejection. It was attributed to the thinnest dense top layer and the highest surface roughness of the PA-15 substrate (confirmed by SEM and AFM data in Figure S8 and Table S1 of Supplementary Materials), which could have an effect on the roughness of a dense selective CMC layer deposited on it [57]. The lowest permeability of the CMC/PAN-15 membrane could be explained by the thickest dense top layer, the lowest surface roughness, and the most hydrophobic surface of the substrate PAN-15 structure (confirmed by SEM, AFM, and contact angle data in Figure S8 and Table S1 of Supplementary Materials) [57]. Thus, the optimal transport properties were observed for the supported CMC/CA-15 membrane, which possessed a high level of permeability (0.23 kg/(m^2^ h atm) for water and 0.22 kg/(m^2^ h atm) for a model solution of heavy metal ions) with the highest rejection coefficients of metal ions (92% of Cd^2+^, 95% of Pb^2+^, 91% of Cu^2+^). This could be due to the most hydrophilic nature of the resulting CA-15 substrate (confirmed by contact angle data in Table S1 of Supplementary Materials), on which a supported CMC-based membrane was prepared. It may facilitate the transfer of water and model solution of heavy metal ions through the membrane, caused by better affinity of this substrate to water with high membrane rejection [57]. Moreover, various types of substrate polymers may affect the crystallinity of the CMC-based selective layer, reflecting the transport properties of supported membranes [57].

**Figure 9:**
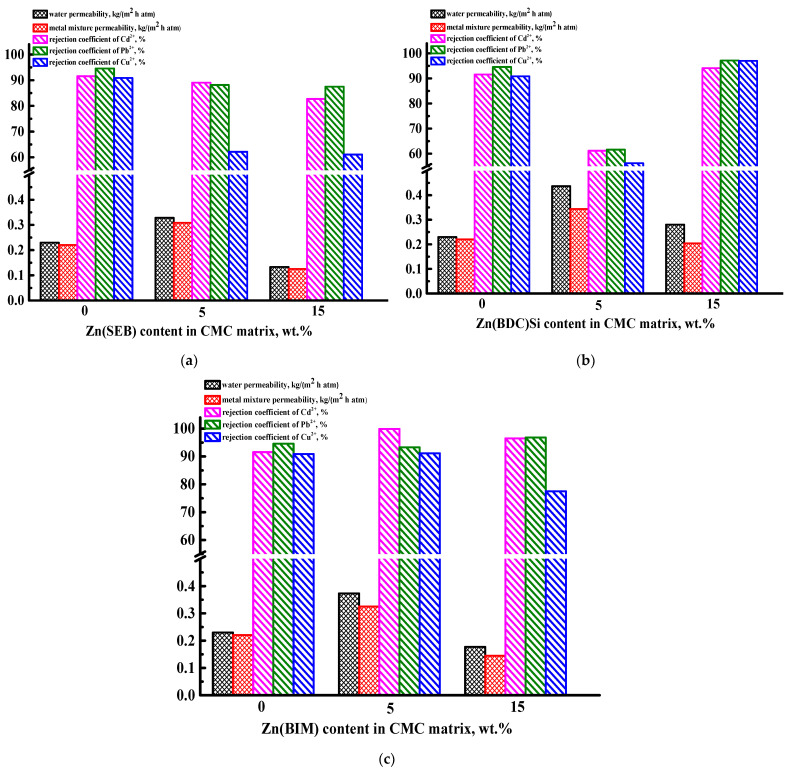
Transport properties in nanofiltration of water and a model solution of heavy metal ions (Cd^2+^, Pb^2+^, Cu^2+^) of the supported (**a**) CMC/CA-15 and CMC + Zn(SEB)/CA-15 membranes, (**b**) CMC/CA-15 and CMC + Zn(BDC)Si/CA-15 membranes, and (**c**) CMC/CA-15 and CMC + Zn(BIM)/CA-15 membranes.

The supported CMC/CA-15 membrane was chosen for further modification with Zn-based MOFs (the introduction of 5 and 15 wt.% Zn(SEB), Zn(BDC)Si, and Zn(BIM) into the CMC layer). The transport properties of cross-linked supported CMC/CA-15 and CMC + Zn-based MOF/CA-15 membranes were studied in nanofiltration of water and a model solution of heavy metal ions (Cd^2+^, Pb^2+^, Cu^2+^) (Figure 9).

It was demonstrated that the introduction of 5 wt.% Zn-based MOFs into the CMC matrix led to the increased permeability of water and model solution of heavy metal ions for the modified supported membranes compared to the pristine CMC/CA-15 membrane. However, a significant reduction in rejection coefficient values for the CMC + Zn(BDC)Si (5%)/CA-15 membrane was observed (61% of Cd^2+^, 62% of Pb^2+^, 56% of Cu^2+^) compared to the other membranes, which could be due to high microporosity and nanoporosity of the Zn(BDC)Si (confirmed by physisorption measurements in Figure S2 of Supplementary Materials). The modification with 15 wt.% Zn(SEB) and Zn(BIM) of the supported membranes led to the decrease in permeability compared to the pristine CMC/CA-15 membrane and rejection coefficients of metal ions compared to CMC + Zn-based MOF (5%)/CA-15 membranes. It may be due to particle agglomerates on the surface of the modified membranes (confirmed by SEM data below), which hindered mass transfer across the membrane, decreasing the permeability and rejection. The CMC + Zn(BDC)Si (15%)/CA-15 membrane had higher permeability of water (0.28 kg/(m^2^ h atm)) and comparatively at the same level of a model solution of heavy metal ions (0.2 kg/(m^2^ h atm)) compared to the pristine CMC/CA-15 membrane, caused by the highest surface roughness among all membranes (confirmed by AFM data below) and structural peculiarity of Zn(BDC)Si particles. Furthermore, it is worth noting that the increase from 5 to 15 wt.% in Zn(BDC)Si content resulted in great growth of rejection coefficients for the CMC + Zn(BDC)Si (15%)/CA-15 membrane compared to all membranes: 94% of Cd^2+^; 97% of Pb^2+^; and 97% of Cu^2+^. It may be explained by the most hydrophobic nature of Zn(BDC)Si among all the MOF particles due to the trimethylsilyl groups inside the Zn(BDC)Si pores (confirmed by X-ray diffraction data in Figures S3 and S4 of Supplementary Materials), which hinder the transfer of metal ions through the membrane increasing the retention capacity. Thus, this supported CMC + Zn(BDC)Si (15%)/CA-15 membrane with optimal transport parameters (with the highest rejection coefficients) was chosen for testing in nanofiltration of water, and a model solution of heavy metal ions (Cd^2+^, Pb^2+^, Cu^2+^) for 6 days (Figure S7b of Supplementary Materials) to confirm its stability and reusability and in nanofiltration of real wastewater from galvanic production (presented in Section 3.3).

#### 3.2.2. Characterization of the Supported CMC-Based Membranes

**Figure 10:**
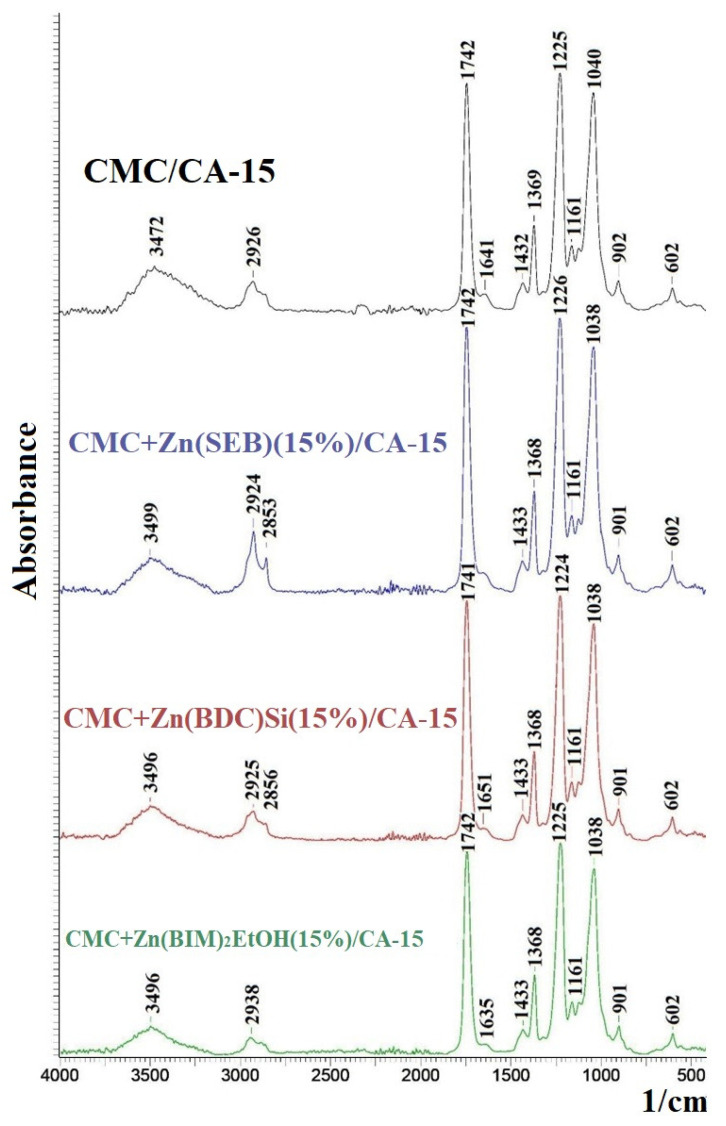
FTIR spectra of the cross-linked supported CMC-based membranes, modified with 15 wt.% Zn-based MOFs.

To explain the nanofiltration performance of cross-linked supported CMC-based membranes, their structural features, and physicochemical properties were studied by FTIR spectroscopy, SEM and AFM microscopies, and contact angle measurements. FTIR spectra of the cross-linked supported CMC-based membranes modified with 15 wt.% Zn-based MOFs are presented in Figure 10.

The FTIR spectrum of the CMC/CA-15 membrane demonstrates characteristic peaks of CMC polymer: at 3472 cm^−1^, related to vibrations of hydroxyl groups; 1641 cm^−1^, corresponded to stretching of the C=O group; 1161 cm^−1^, referred to C–O–C [23]. The weak peak at about 1046 cm^−1^ refers to the stretching vibration of C–O–C bonds formed as a result of the reaction between CMC and GA, confirming the cross-linking of polymer chains [23,58]. The absence of the peak at 1720 cm^−1^, related to the C=O stretching vibration of the aldehyde group of the GA, indicates the absence of an unreacted cross-linking agent [23]. The introduction of Zn-based MOFs into the CMC matrix practically did not change the FTIR spectra of the modified membranes. However, the peak at 3472 cm^−1^, related to hydroxyl groups, decreases in its intensity and shifts to 3499, 3496, and 3496 cm^−1^ during the modification with 15 wt.% Zn(SEB), Zn(BDC)Si, and Zn(BIM), respectively. These changes may testify to the formation of hydrogen bonds between Zn-based MOFs and CMC [16,48]. The XRD profiles of the CMC membranes modified with Zn(SEB), Zn(BDC)Si, and Zn(BIM) were also compared with the XRD profiles of synthesized Zn-based MOFs to determine the structural integrity of modifiers in membranes (Figure S6 of Supplementary Materials).

**Figure 11:**
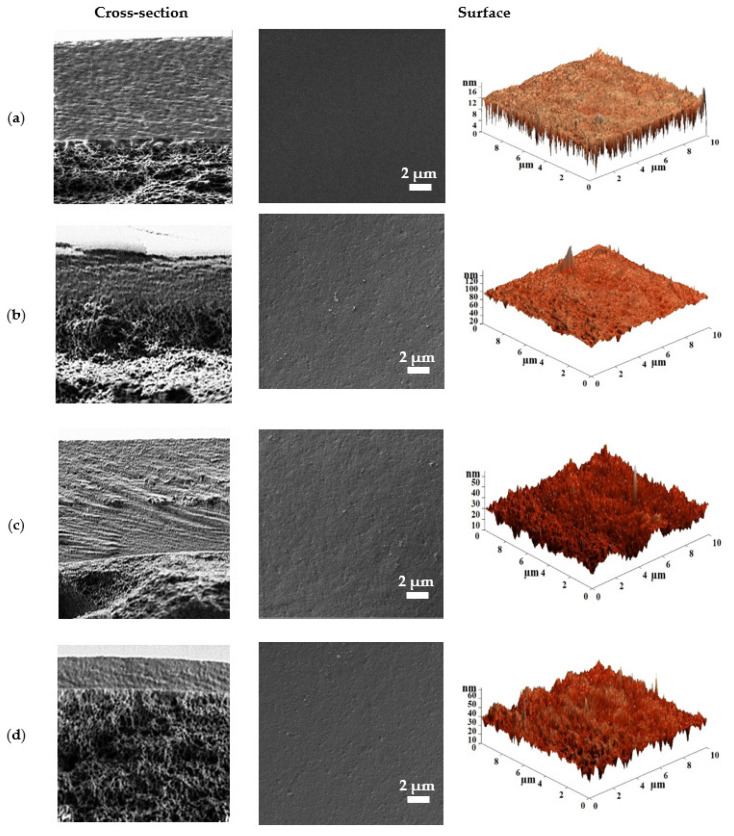
Cross-sectional and surface SEM micrographs and surface AFM images of (**a**) CMC/CA-15, (**b**) CMC + Zn(SEB) (15%)/CA-15, (**c**) CMC + Zn(BDC)Si (15%)/CA-15, and (**d**) CMC + Zn(BIM) (15%)/CA-15 membranes.

The cross-sectional and surface morphology of the cross-linked supported CMC-based membranes were studied by SEM and AFM (Figure 11).

All cross-sectional SEM micrographs of the supported CMC-based membranes demonstrate two regions: (1) a continuous dense selective CMC-based layer with good adhesion to the surface of (2) the porous CA-15 substrate. No leakage of the polymer solution and composite into the substrate pores was observed [47]. The thickness of the thin dense selective layer varied depending on the introduced modifier: 5 µm for CMC/CA-15 membrane; 1.5 µm for CMC + Zn(SEB) (15%)/CA-15 membrane; 10 µm for CMC + Zn(BDC)Si (15%)/CA-15 membrane; and 660 nm for CMC + Zn(BIM) (15%)/CA-15 membrane. This may also be related to the size of the introduced MOF particle into the CMC matrix. For example, the maximum thickness of the thin dense selective layer was observed for CMC + Zn(BDC)Si (15%)/CA-15 membrane, the thickening of which may be due to the introduction of Zn(BDC)Si with the largest particle size (confirmed by SEM data in Figure S5 of Supplementary Materials). The introduction of Zn-based MOFs into the CMC matrix also changed the surface morphology of the modified membranes. For the CMC/CA-15 membrane (Figure 11a), a smooth surface structure without any defects was noted, while for the modified membranes, particle agglomerates were observed on the membrane surface [43]. The maximum surface roughness was observed for the CMC + Zn(BDC)Si (15%)/CA-15 membrane (Figure 11c), which resulted in the highest permeability in nanofiltration compared to all membranes. All these morphology changes of the modified membranes were due to the structural and shape peculiarity of Zn-based MOFs (confirmed in Section S1 of Supplementary Materials). Based on the AFM images, the average surface roughness of the supported cross-linked CMC-based membranes was calculated (Table 2). To evaluate the hydrophilic–hydrophobic balance changes of the membrane surface, the contact angles of water were measured and presented in Table 2.

The obtained average surface roughness data are in agreement with SEM micrographs (Figure 11). It was demonstrated that the introduction of 15 wt.% Zn-based MOFs into the CMC matrix led to an increase in the surface roughness of the modified membranes due to particle agglomeration. The maximum surface roughness was observed for the CMC + Zn(BDC)Si (15%)/CA-15 membrane, which resulted in the highest permeability in nanofiltration compared to modified membranes. The contact angle of water for the CMC/CA-15 membrane is equal to 54°, which is close to the previously obtained data in the works [21,23]. For the modified membranes, a decrease in the contact angle data was observed, indicating an increase in the membrane surface hydrophilicity [22]. It was attributed to the hydrophilic Zn-MOF nature [38]. The mechanical properties of the CMC/CA-15 and CMC + Zn(BDC)Si (15%)/CA-15 membranes were also evaluated. For these supported membranes, the values of maximum tension and elongation were comparable (0.70 MPa and 1.92 mm, respectively). The mechanical properties of supported membranes were determined by the CA-15 substrate characteristics due to the large difference in the thickness of the thin dense selective CMC-based layer (10 μm) and a porous CA-15 substrate with a thickness of 100 μm.

### 3.3. Investigation of Membrane Performance in Nanofiltration of Wastewater from Galvanic Production

To assess the prospects for application in industry, the transport properties of the developed modified membranes with improved properties (porous CA-12/Zn(SEB) (1%) and supported cross-linked CMC + Zn(BDC)Si (15%)/CA-15 membranes) were studied in nanofiltration of a real object—wastewater from galvanic production (LLC “Galvanik”, St. Petersburg, Russia), containing heavy metal ions (Cu^2+^, Cd^2+^, C^r3+^, Ni^2+^, Zn^2+^). The pristine porous CA-12 and supported cross-linked CMC/CA-15 membranes were also tested for comparison. The transport parameters are presented in Table 3.

It was found that the modification of pristine membranes with Zn-based MOFs led to an increase in both rejection coefficients and permeability of wastewater. The CA-12/Zn(SEB) (1%) membrane had an increase in rejection coefficients of heavy metal ions up to 26% for Cu^2+^, 8% for Cd^2+^, 6% for Cr^3+^, 8% for Ni^2+,^ and 7% for Zn^2+^ and the highest permeability of wastewater to 49 kg/(m^2^ h atm), while the highest rejection coefficients of heavy metal ions of 80% for Cu^2+^, 93% for Cd^2+^, 79% for Cr^3+^, 91% for Ni^2+,^ and 95% for Zn^2+^ with 0.1 kg/(m^2^ h atm) permeability were observed for the CMC + Zn(BDC)Si (15%)/CA-15 membrane. This trend is explained by the structure of the developed membranes: a porous membrane (CA-12/Zn(SEB) (1%)) has the least resistance to the mass transfer of components, causing the highest permeability with low rejection. When a selective layer in a supported membrane provides a high rejection level, retarding the membrane permeability due to the dense packing of the top polymer selective layer.

The performance comparison of the developed modified membranes with improved properties (porous CA-12/Zn(SEB) (1%) and supported cross-linked CMC + Zn(BDC)Si (15%)/CA-15 membranes) with described in the literature membranes for nanofiltration of heavy metal ions solutions was also carried out (Tables S2 and S3 of Supplementary Materials). It was demonstrated that the porous CA-12/Zn(SEB) (1%) membrane had improved permeability compared to the porous CA-based membranes, while CMC + Zn(BDC)Si (15%)/CA-15 membrane exhibited improved rejection coefficients of heavy metal ions compared to nonporous biopolymer membranes. It should also be emphasized that there are no studies of the CA and CMC-based membranes in the nanofiltration of solutions of a heavy metal ions mixture. Thus, a high-performance porous CA-12/Zn(SEB) (1%) and a highly selective supported CMC + Zn(BDC)Si (15%)/CA-15 membranes have been developed for water treatment by nanofiltration, which one or the other can be used in the industrial future, depending on the separation tasks.

## 4. Conclusions

Two types of novel highly-efficient sustainable nanofiltration membranes for water treatment from heavy metal ions were developed: porous CA membranes and supported CMC membranes. The improvement of the transport properties of the developed membranes was achieved through synthesized for the first time Zn-based MOF (Zn(SEB), Zn(BDC)Si, and Zn(BIM)) modifications due to their porous structure, hydrophilic properties, and different particle shapes.

For porous membranes, the influence of casting solution concentration variation for CA membrane preparation was studied in the nanofiltration of water and a model Cu^2+^ solution. The CA-12 membrane (prepared from 12 wt.%) was chosen for the modification with 1 wt.% Zn-based MOFs due to the highest permeability, caused by the highest porosity, surface roughness, and hydrophilicity. The CA-12/Zn-based MOF (1 wt.%) membranes had increased permeability and rejection coefficients in nanofiltration of water, a model Cu^2+^, and heavy metal ion (Cd^2+^, Pb^2+^, Cu^2+^) solutions compared to pristine one due to increased inner membrane porosity structure attributed to the peculiarity of MOF structure, surface roughness, and hydrophilicity. The porous CA-12/Zn(SEB) (1%) membrane had the best transport properties: 144 and 105 kg/(m^2^h atm) water and heavy metal ion solution permeability, respectively, with rejection coefficients for Cd^2+^, Pb^2+^, Cu^2+^—63, 78, and 47%, respectively.

The nanofiltration-supported CMC-based membranes were developed by the deposition of a thin dense CMC layer onto the developed porous CA, PA, and PAN membranes (applied as substrates). The optimal substrate for the supported CMC membrane was a porous membrane based on CA prepared from a 15 wt.% casting solution, as this membrane had the best compromise of permeability and heavy metal ion rejection. Among the supported CMC/CA-15 membranes modified with 5 and 15 wt.% Zn-based MOFs, the CMC + Zn(BDC)Si (15%)/CA-15 membrane had optimal properties in nanofiltration due to structural peculiarity of Zn(BDC)Si particles resulted to the highest surface roughness: 0.28 and 0.2 kg/(m^2^ h atm) water and solution permeability, respectively, and the highest level of rejection coefficients (94% of Cd^2+^, 97% of Pb^2+^, 97% of Cu^2+^).

To confirm the perspective industrial application, the porous CA-12/Zn(SEB) (1%) and supported cross-linked CMC + Zn(BDC)Si (15%)/CA-15 membranes with improved properties were studied in nanofiltration of a real object—wastewater from galvanic production. It was demonstrated that these developed membranes could be used in the industry for water treatment from heavy metal ions depending on the separation tasks.

## Figures and Tables

**Table 1:** The average surface roughness, contact angle, and total porosity for the developed porous CA-based membranes.

Membrane	Ra, nm	Contact Angle, °	Total Porosity, %	Porosity Over Weight, cm^3^/g
CA-12	3.16	30	95.8	4.5
CA-15	1.91	31	94.6	2.2
CA-17	1.65	31	94.4	1.9
CA-20	1.60	33	91.8	1.2
CA-12/Zn(SEB) (1%)	2.97	26	89.1	3.4
CA-12/Zn(BDC)Si (1%)	3.29	27	87.0	2.8
CA-12/Zn(BIM) (1%)	3.08	28	87.6	2.9

**Table 2:** The average surface roughness and contact angle for the developed supported cross-linked CMC-based membranes.

Membrane	Ra, nm	Contact Angle of Water, °
CMC/CA-15	1.1	54
CMC + Zn(SEB) (15%)/CA-15	2.9	45
CMC + Zn(BDC)Si (15%)/CA-15	3.4	50
CMC + Zn(BIM) (15%)/CA-15	3.1	49

**Table 3:** Transport properties of membranes in nanofiltration of wastewater from galvanic production containing heavy metal ions (Cu^2+^, Cd^2+^, Cr^3+^, Ni^2+^, Zn^2+^).

Membrane	Permeability, kg/(m^2^ h atm)	Rejection Coefficient, %				
		Cu^2+^	Cd^2+^	Cr^3+^	Ni^2+^	Zn^2+^
CA-12	45	0	1	2	0	1
CA-12/Zn(SEB) (1%)	49	26	8	6	8	7
CMC/CA-15	0.06	23	82	77	38	50
CMC + Zn(BDC)Si (15%)/CA-15	0.10	80	93	79	91	95

## Data Availability

The data presented in this study are available on request from the corresponding author.

## Supplementary Materials

The following supporting information can be downloaded at: https://www.mdpi.com/article/10.3390/polym15061341/s1, The following supporting information (synthesis and characterization of Zn-based MOFs and the characterization of the developed membranes and substrates) is available free of charge via the Internet as a file “Supplementary Materials”, Figure S1: Structure of organic ligands; Figure S2: Nitrogen adsorption–desorption isotherms on Zn-based MOFs: (a) Zn(SEB); (b) Zn(BDC)Si; (c) Zn(BIM)_2_EtOH; Figure S3: XRD profile of Zn-based MOFs with simulated profile overlay: (a) Zn(SEB) (black line) and poly[([mu]4-decanedio-ato)cobalt(II)] (blue line); (b) Zn(BDC)Si (black line) and zinc terephthalate (Zn(BDC), MOF-5) (blue line); and (c) Zn(BIM) (black line) and (Co)ZIF-9 (blue line); Figure S4: Structures of (a) Zn(SEB), (b) Zn(BDC)Si, and (c) Zn(BIM); Figure S5: SEM micrographs at different magnifications of Zn-based MOFs; Figure S6: Shifted XRD profiles of (a) the porous CA-12 (blue line) and dense CMC (red line) membranes, (b) the modified with Zn(SEB) porous CA-12 (blue line) and dense CMC (red line) membranes and Zn(SEB) (black line), (c) the modified with Zn(BDC)Si porous CA-12 (blue line) and dense CMC (red line) membranes and Zn(BDC)Si (black line), and (d) the modified with Zn(BIM) porous CA-12 (blue line) and dense CMC (red line) membranes and Zn(BIM) (black line); Figure S7: The dependence of the permeability of water and a model solution of heavy metal ions (Cd^2+^, Pb^2+^, Cu^2+^) on the number of days of nanofiltration experiments for (a) porous CA-12 and CA-12/Zn(SEB) (1%) membranes, (b) supported cross-linked CMC/CA-15 and CMC + Zn(BDC)Si (15%)/CA-15 membranes; Figure S8: Cross-sectional SEM micrographs at different magnifications and surface AFM images of (a) CA-15, (b) PA-15, and (c) PAN-15 substrates; Table S1: The average surface roughness, contact angle and total porosity for the developed porous substrates; Table S2: Comparison of transport properties for the porous CA-based membranes in nanofiltration of heavy metal ions; Table S3: Comparison of transport properties for the nonporous biopolymer membranes in nanofiltration of heavy metal ions.

## References

[1] Meng Y., Shu L., Liu L., Wu Y., Xie L.-H., Zhao M.-J., Li J.-R. (2019). A high-flux mixed matrix nanofiltration membrane with highly water-dispersible MOF crystallites as filler. J. Membr. Sci..

[2] Gnanasekaran G., Balaguru S., Arthanareeswaran G., Das D.B. (2019). Removal of hazardous material from wastewater by using metal organic framework (MOF) embedded polymeric membranes. Sep. Sci. Technol..

[3] Luo X.-P., Fu S.-Y., Du Y.-M., Guo J.-Z., Li B. (2017). Adsorption of methylene blue and malachite green from aqueous solution by sulfonic acid group modified MIL-101. Microporous Mesoporous Mater..

[4] Bhadra B.N., Ahmed I., Jhung S.H. (2016). Remarkable adsorbent for phenol removal from fuel: Functionalized metal–organic framework. Fuel.

[5] Sabzehmeidani M.M., Mahnaee S., Ghaedi M., Heidari H., Roy V.A.L. (2021). Carbon based materials: A review of adsorbents for inorganic and organic compounds. Mater. Adv..

[6] Obey G., Adelaide M., Ramaraj R. (2022). Biochar derived from non-customized matamba fruit shell as an adsorbent for wastewater treatment. J. Bioresour. Bioprod..

[7] Saad E.M., Elshaarawy R.F., Mahmoud S.A., El-Moselhy K.M. (2021). New *Ulva lactuca* Algae Based Chitosan Bio-composites for Bioremediation of Cd(II) Ions. J. Bioresour. Bioprod..

[8] Qasem N.A.A., Mohammed R.H., Lawal D.U. (2021). Removal of heavy metal ions from wastewater: A comprehensive and critical review. NPJ Clean Water.

[9] Kuzminova A., Dmitrenko M., Zolotarev A., Korniak A., Poloneeva D., Selyutin A., Emeline A., Yushkin A., Foster A., Budd P. (2021). Novel Mixed Matrix Membranes Based on Polymer of Intrinsic Microporosity PIM-1 Modified with Metal-Organic Frameworks for Removal of Heavy Metal Ions and Food Dyes by Nanofiltration. Membranes.

[10] Idress H., Zaidi S.Z.J., Sabir A., Shafiq M., Khan R.U., Harito C., Hassan S., Walsh F.C. (2021). Cellulose acetate based Complexation-NF membranes for the removal of Pb(II) from waste water. Sci. Rep..

[11] Mondal P., Purkait M.K. (2018). Green synthesized iron nanoparticles supported on pH responsive polymeric membrane for nitrobenzene reduction and fluoride rejection study: Optimization approach. J. Clean. Prod..

[12] Otvagina K., Penkova A., Dmitrenko M., Kuzminova A., Sazanova T., Vorotyntsev A., Vorotyntsev I. (2019). Novel Composite Membranes Based on Chitosan Copolymers with Polyacrylonitrile and Polystyrene: Physicochemical Properties and Application for Pervaporation Dehydration of Tetrahydrofuran. Membranes.

[13] Mishra A.K. (2016). Smart Materials for Waste Water Applications.

[14] Su J., Yang Q., Teo J.F., Chung T.-S. (2010). Cellulose acetate nanofiltration hollow fiber membranes for forward osmosis processes. J. Membr. Sci..

[15] Loeb S., Sourirajan S. (1963). Sea Water Demineralization by Means of an Osmotic Membrane. Saline Water Conversion—II.

[16] Alhalili Z., Romdhani C., Chemingui H., Smiri M. (2021). Removal of dithioterethiol (DTT) from water by membranes of cellulose acetate (AC) and AC doped ZnO and TiO_2_ nanoparticles. J. Saudi Chem. Soc..

[17] Rasool M.A., Vankelecom I.F.J. (2021). Preparation of full-bio-based nanofiltration membranes. J. Membr. Sci..

[18] Rasool M.A., Van Goethem C., Vankelecom I.F.J. (2020). Green preparation process using methyl lactate for cellulose-acetate-based nanofiltration membranes. Sep. Purif. Technol..

[19] Ounifi I., Ursino C., Santoro S., Chekir J., Hafiane A., Figoli A., Ferjani E. (2020). Cellulose acetate nanofiltration membranes for cadmium remediation. J. Membr. Sci. Res..

[20] Shao L.-L., An Q.-F., Ji Y.-L., Zhao Q., Wang X.-S., Zhu B.-K., Gao C.-J. (2014). Preparation and characterization of sulfated carboxymethyl cellulose nanofiltration membranes with improved water permeability. Desalination.

[21] Jabbarvand Behrouz S., Khataee A., Safarpour M., Arefi-Oskoui S., Woo Joo S. (2021). Carboxymethyl cellulose/polyethersulfone thin-film composite membranes for low-pressure desalination. Sep. Purif. Technol..

[22] Miao J., Zhang R., Bai R. (2015). Poly (vinyl alcohol)/carboxymethyl cellulose sodium blend composite nanofiltration membranes developed via interfacial polymerization. J. Membr. Sci..

[23] Zhang Y., Yu C., Lü Z., Yu S. (2016). Modification of polysulfone ultrafiltration membrane by sequential deposition of cross-linked poly(vinyl alcohol) (PVA) and sodium carboxymethyl cellulose (CMCNa) for nanofiltration. Desalin. Water Treat..

[24] Chen Q., Yu P., Huang W., Yu S., Liu M., Gao C. (2015). High-flux composite hollow fiber nanofiltration membranes fabricated through layer-by-layer deposition of oppositely charged crosslinked polyelectrolytes for dye removal. J. Membr. Sci..

[25] Yu S., Zheng Y., Zhou Q., Shuai S., Lü Z., Gao C. (2012). Facile modification of polypropylene hollow fiber microfiltration membranes for nanofiltration. Desalination.

[26] Yu S., Chen Z., Cheng Q., Lü Z., Liu M., Gao C. (2012). Application of thin-film composite hollow fiber membrane to submerged nanofiltration of anionic dye aqueous solutions. Sep. Purif. Technol..

[27] Zhao Q., Ji Y.-L., Wu J.-K., Shao L.-L., An Q.-F., Gao C.-J. (2014). Polyelectrolyte complex nanofiltration membranes: Performance modulation via casting solution pH. RSC Adv..

[28] Ji Y., An Q., Zhao Q., Chen H., Qian J., Gao C. (2010). Fabrication and performance of a new type of charged nanofiltration membrane based on polyelectrolyte complex. J. Membr. Sci..

[29] Dmitrenko M., Chepeleva A., Liamin V., Kuzminova A., Mazur A., Semenov K., Penkova A. (2022). Novel PDMS-b-PPO Membranes Modified with Graphene Oxide for Efficient Pervaporation Ethanol Dehydration. Membranes.

[30] Plisko T.V., Bildyukevich A.V., Burts K.S., Ermakov S.S., Penkova A.V., Kuzminova A.I., Dmitrenko M.E., Hliavitskaya T.A., Ulbricht M. (2020). One-Step Preparation of Antifouling Polysulfone Ultrafiltration Membranes via Modification by a Cationic Polyelectrolyte Based on Polyacrylamide. Polymers.

[31] Dmitrenko M., Liamin V., Kuzminova A., Mazur A., Lahderanta E., Ermakov S., Penkova A. (2020). Novel Mixed Matrix Sodium Alginate–Fullerenol Membranes: Development, Characterization, and Study in Pervaporation Dehydration of Isopropanol. Polymers.

[32] Dmitrenko M., Kuzminova A., Zolotarev A., Liamin V., Plisko T., Burts K., Bildyukevich A., Ermakov S., Penkova A. (2021). Novel High Flux Poly(m-phenylene isophtalamide)/TiO_2_ Membranes for Ultrafiltration with Enhanced Antifouling Performance. Polymers.

[33] Dmitrenko M., Kuzminova A., Zolotarev A., Markelov D., Komolkin A., Loginova E., Plisko T., Burts K., Bildyukevich A., Penkova A. (2022). Modification strategies of polyacrylonitrile ultrafiltration membrane using TiO_2_ for enhanced antifouling performance in water treatment. Sep. Purif. Technol..

[34] Dmitrenko M.E., Kuzminova A.I., Zolotarev A.A., Korniak A.S., Ermakov S.S., Su R., Penkova A.V. (2022). Novel mixed matrix membranes based on polyelectrolyte complex modified with fullerene derivatives for enhanced pervaporation and nanofiltration. Sep. Purif. Technol..

[35] Gribanova E.V., Larionov M.I. (2014). Application of contact angle dependence on ph for estimation of acid-base properties of oxide surfaces. Vestn. St. -Petersbg. Univ. Ser. 4. PHYSICS Chem..

[36] Plisko T.V., Liubimova A.S., Bildyukevich A.V., Penkova A.V., Dmitrenko M.E., Mikhailovskii V.Y., Melnikova G.B., Semenov K.N., Doroshkevich N.V., Kuzminova A.I. (2018). Fabrication and characterization of polyamide-fullerenol thin film nanocomposite hollow fiber membranes with enhanced antifouling performance. J. Membr. Sci..

[37] Dmitrenko M.E., Penkova A.V., Atta R.R., Zolotarev A.A., Plisko T.V., Mazur A.S., Solovyev N.D., Ermakov S.S. (2019). The development and study of novel membrane materials based on polyphenylene isophthalamide—Pluronic F127 composite. Mater. Des..

[38] Shukla A.K., Alam J., Ali F.A.A., Alhoshan M. (2020). A highly permeable zinc-based MOF/polyphenylsulfone composite membrane with elevated antifouling properties. Chem. Commun..

[39] Shukla A.K., Alam J., Alhoshan M.S., Ali F.A.A., Mishra U., Hamid A.A. (2021). Thin-Film Nanocomposite Membrane Incorporated with Porous Zn-Based Metal–Organic Frameworks: Toward Enhancement of Desalination Performance and Chlorine Resistance. ACS Appl. Mater. Interfaces.

[40] Elrasheedy A., Nady N., Bassyouni M., El-Shazly A. (2019). Metal Organic Framework Based Polymer Mixed Matrix Membranes: Review on Applications in Water Purification. Membranes.

[41] Jun B.-M., Al-Hamadani Y.A.J., Son A., Park C.M., Jang M., Jang A., Kim N.C., Yoon Y. (2020). Applications of metal-organic framework based membranes in water purification: A review. Sep. Purif. Technol..

[42] Elsaidi S.K., Venna S.R., Mohamed M.H., Gipple M.J., Hopkinson D.P. (2020). Dual-Layer MOF Composite Membranes with Tuned Interface Interaction for Postcombustion Carbon Dioxide Separation. Cell Rep. Phys. Sci..

[43] Kuzminova A., Dmitrenko M., Zolotarev A., Myznikov D., Selyutin A., Su R., Penkova A. (2022). Pervaporation Polyvinyl Alcohol Membranes Modified with Zr-Based Metal Organic Frameworks for Isopropanol Dehydration. Membranes.

[44] Kuzminova A., Dmitrenko M., Mazur A., Ermakov S., Penkova A. (2021). Novel Pervaporation Membranes Based on Biopolymer Sodium Alginate Modified by FeBTC for Isopropanol Dehydration. Sustainability.

[45] Gu Q., Ng H.Y., Zhao D., Wang J. (2020). Metal–Organic Frameworks (MOFs)-boosted filtration membrane technology for water sustainability. APL Mater..

[46] Wongsasulak S., Patapeejumruswong M., Weiss J., Supaphol P., Yoovidhya T. (2010). Electrospinning of food-grade nanofibers from cellulose acetate and egg albumen blends. J. Food Eng..

[47] Dmitrenko M., Chepeleva A., Liamin V., Mazur A., Semenov K., Solovyev N., Penkova A. (2022). Novel Mixed Matrix Membranes Based on Polyphenylene Oxide Modified with Graphene Oxide for Enhanced Pervaporation Dehydration of Ethylene Glycol. Polymers.

[48] Sui X., Shao C., Liu Y. (2007). Photoluminescence of polyethylene oxide–ZnO composite electrospun fibers. Polymer.

[49] Tan X., Rodrigue D. (2019). A Review on Porous Polymeric Membrane Preparation. Part I: Production Techniques with Polysulfone and Poly (Vinylidene Fluoride). Polymers.

[50] Zhang Y., Jia H., Wang Q., Ma W., Yang G., Xu S., Li S., Su G., Qu Y., Zhang M. (2021). Optimization of a MOF Blended with Modified Polyimide Membrane for High-Performance Gas Separation. Membranes.

[51] Yang Z., Ao D., Guo X., Nie L., Qiao Z., Zhong C. (2021). Preparation and characterization of small-size amorphous MOF mixed matrix membrane. Sep. Purif. Technol..

[52] Taghipour A., Rahimpour A., Rastgar M., Sadrzadeh M. (2022). Ultrasonically synthesized MOFs for modification of polymeric membranes: A critical review. Ultrason. Sonochem..

[53] Shahid S., Nijmeijer K., Nehache S., Vankelecom I., Deratani A., Quemener D. (2015). MOF-mixed matrix membranes: Precise dispersion of MOF particles with better compatibility via a particle fusion approach for enhanced gas separation properties. J. Membr. Sci..

[54] Qiu C., Panwisawas C., Ward M., Basoalto H.C., Brooks J.W., Attallah M.M. (2015). On the role of melt flow into the surface structure and porosity development during selective laser melting. Acta Mater..

[55] Burts K.S., Plisko T.V., Bildyukevich A.V., Penkova A.V., Pratsenko S.A. (2021). Modification of polysulfone ultrafiltration membranes using block copolymer Pluronic F127. Polym. Bull..

[56] Xu T.-C., Wang C.-S., Hu Z.-Y., Zheng J.-J., Jiang S.-H., He S.-J., Hou H.-Q. (2022). High Strength and Stable Proton Exchange Membrane Based on Perfluorosulfonic Acid/Polybenzimidazole. Chin. J. Polym. Sci..

[57] Dmitrenko M., Kuzminova A., Zolotarev A., Ermakov S., Roizard D., Penkova A. (2020). Enhanced pervaporation properties of PVA-based membranes modified with polyelectrolytes. application to IPA dehydration. Polymers.

[58] Liu Y., Zhu M., Zhao Q., An Q., Qian J., Lee K., Lai J. (2011). The chemical crosslinking of polyelectrolyte complex colloidal particles and the pervaporation performance of their membranes. J. Membr. Sci..

