# Supplementary material for: Nanofiltration Mixed Matrix Membranes from Cellulose Modified with Zn-Based Metal–Organic Frameworks for the Enhanced Water Treatment from Heavy Metal Ions
Source: Polymers (Basel). 2023 Mar 7;15(6):1341. doi: 10.3390/polym15061341 (PMC10052156; doi:10.3390/polym15061341)
Supplement: Supplementary file 1 [file polymers-15-01341-s001.zip › polymers-2221198-supplementary.pdf]

SUPPLEMENTARY MATERIAL

Nanofiltration mixed matrix membranes from cellulose modified with Zn-based metal organic frameworks for the enhanced water treatment from heavy metal ions

Mariia Dmitrenko[a,*], Anna Kuzminova[a], Andrey Zolotarev[a], Artem Selyutin[a], Sergey Ermakov[a], Anastasia Penkova[a]

[a] *St. Petersburg State University, 7/9 Universitetskaya nab., St. Petersburg 199034, Russia*

*Corresponding author: m.dmitrienko@spbu.ru; Tel.: +7-(812)-363-60-00 (ext. 3367)

S1. Synthesis and characterization of Zn-MOFs particles

Materials

To obtain a series of Zn-based metal organic frameworks (MOFs), the following reagents were used: zinc acetate anhydrous (99.99%, Alfa Aesar), benzimidazole (99.9%, ChemicalLine), sebacic acid (99%, Vekton), 2-trimethylsilylterephthalic acid (TSTA) obtained at the Institute of Macromolecular Compounds Russian Academy of Sciences according to the method [1], glacial acetic acid (99.9%, Vekton), ethanol.

Synthesis of Zn-MOFs particles

To obtain Zn-based MOFs as Zn(SEB) (sebacic acid as a ligand), Zn(BDC)Si (2-trimethylsilylterephthalic acid), Zn(BIM) (benzimidazole as a ligand), anhydrous zinc acetate and the ligand were dissolved in ethanol in a molar ratio of 1:1 with the addition of glacial acetic acid. The structure of organic ligands is presented in Figure S1. When the solution was left to stand, a white precipitate slowly formed over two days, which was filtered off under reduced pressure and washed with an aqueous solution of acetic acid and ethanol. The filter cake was dried in a stream of air. The resulting Zn-based MOFs were subjected to thermochemical activation in a vacuum oven at 60°C.

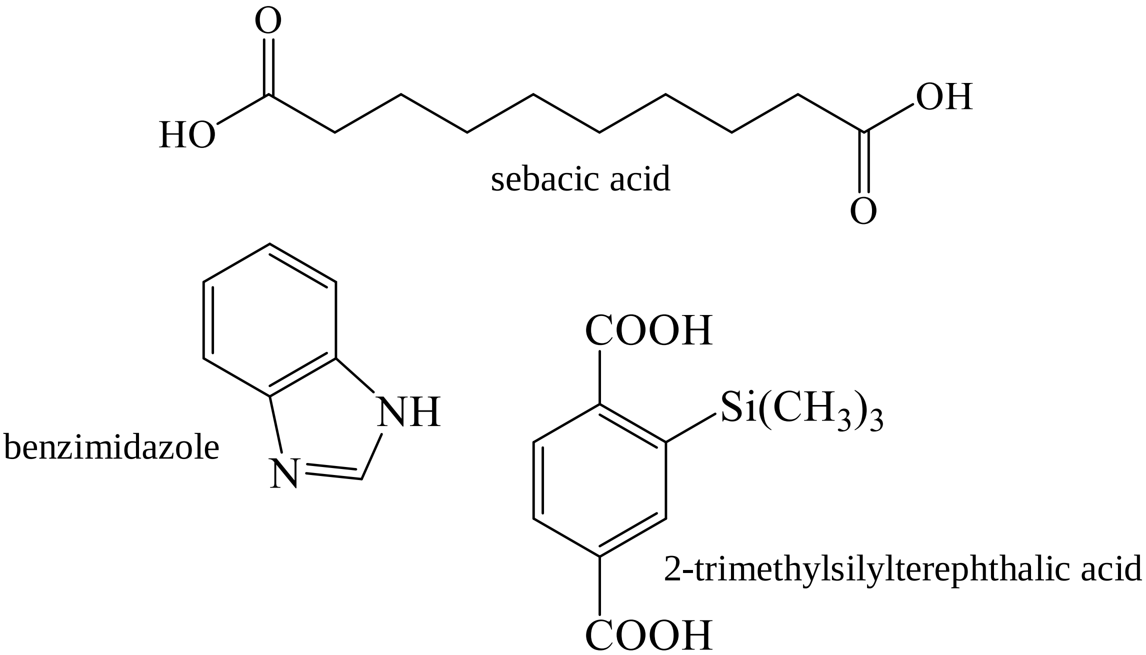

Figure S1. Structure of organic ligands.

Characterization of Zn-MOFs particles

Physisorption measurements with nitrogen were performed at 77K with ASAP 2020 MP analyzer (Micromeritics, USA). The specific surface area for the obtained Zn(SEB) MOF was calculated by the BET method [2], which was equal to 78 m^2/g (Figure S2a). Determination of the pore size distribution by the BJH method with Harkins-Jura and Faas correction [3,4] gave an average pore size of 20-40 nm with a total area of 75 m^2/g. The specific surface area for the obtained Zn(BDC)Si was calculated by the Langmuir method and equal to 294 m^2/g (Figure S2b). The determination of the specific surface by the BET method turned out to be impossible due to the negative values of the interaction constant. This could be due to the presence of high microporosity and nanoporosity of the sample. The specific surface area for the Zn(BIM) calculated by the BET method was 7 m^2/g (Figure S2c). There was the absence of porosity, which could be explained by the effect of "breathing" in such MOF. Such MOF structures are very good sorbents, the pores in the structure are quickly occupied by gases and vapors from the atmosphere. During sample preparation (especially for Zn(SEB) and Zn(BIM)) for measuring the specific surface area, a large number of pores are occupied and not available for the absorbate gas (nitrogen). Thus, the resulting values of the specific surface area obtained by the BET method were underestimated. But this fact is not essential for this study, since the obtained Zn-based MOF modified membranes are not used for gas separation. At the same time, the permeability of such coordination polymers remains high for the flow of liquids.

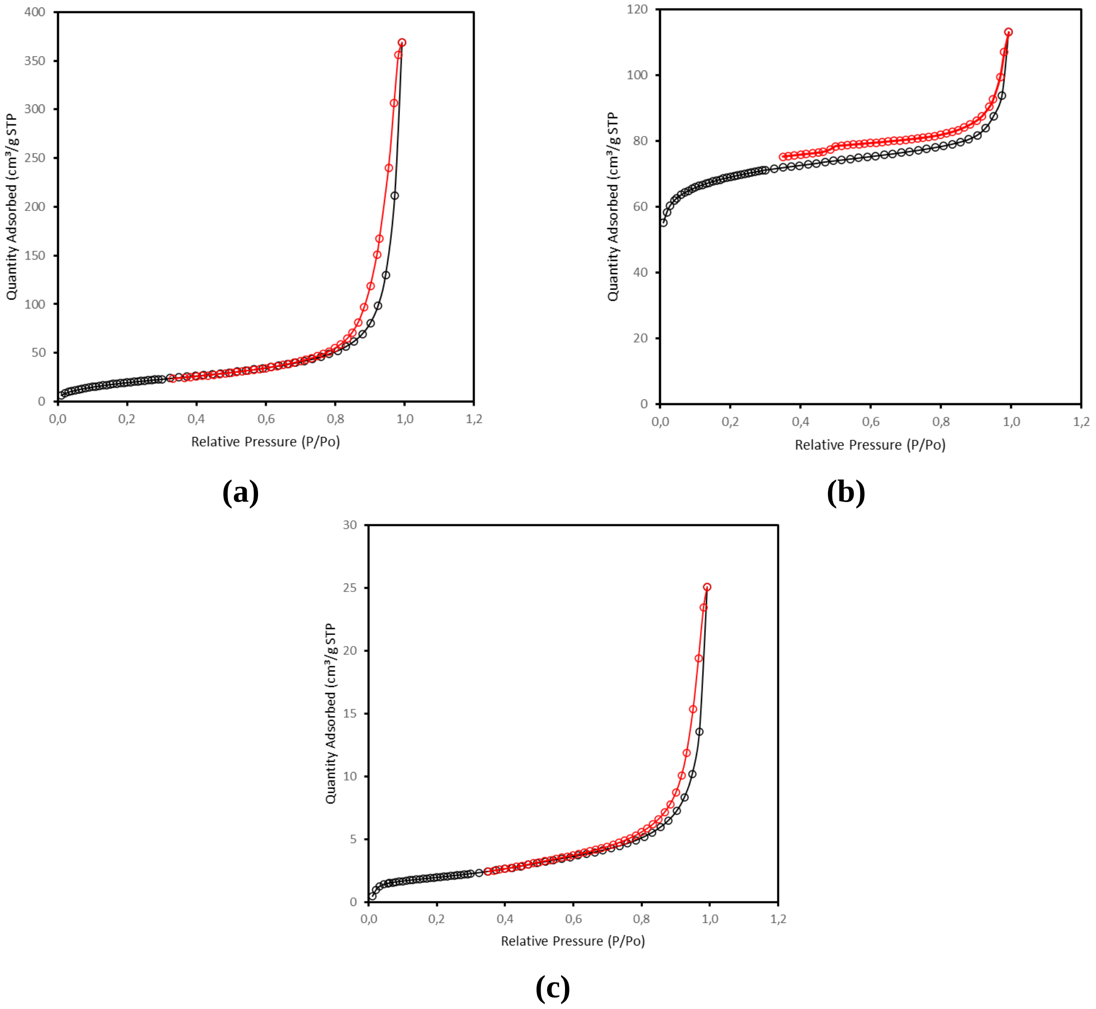

Figure S2. Nitrogen adsorption-desorption isotherms on Zn-based MOFs: (a) Zn(SEB), (b) Zn(BDC)Si, (c) Zn(BIM).

The phase composition and crystal structure of the resulting Zn-based MOFs were determined using «MiniFlexII» powder diffractometer (Rigaku, Japan) with CuKα-radiation. X-

ray fluorescence (XRF) profiles of Zn-based MOFs with simulated profiles overlay are presented in Figure S3.

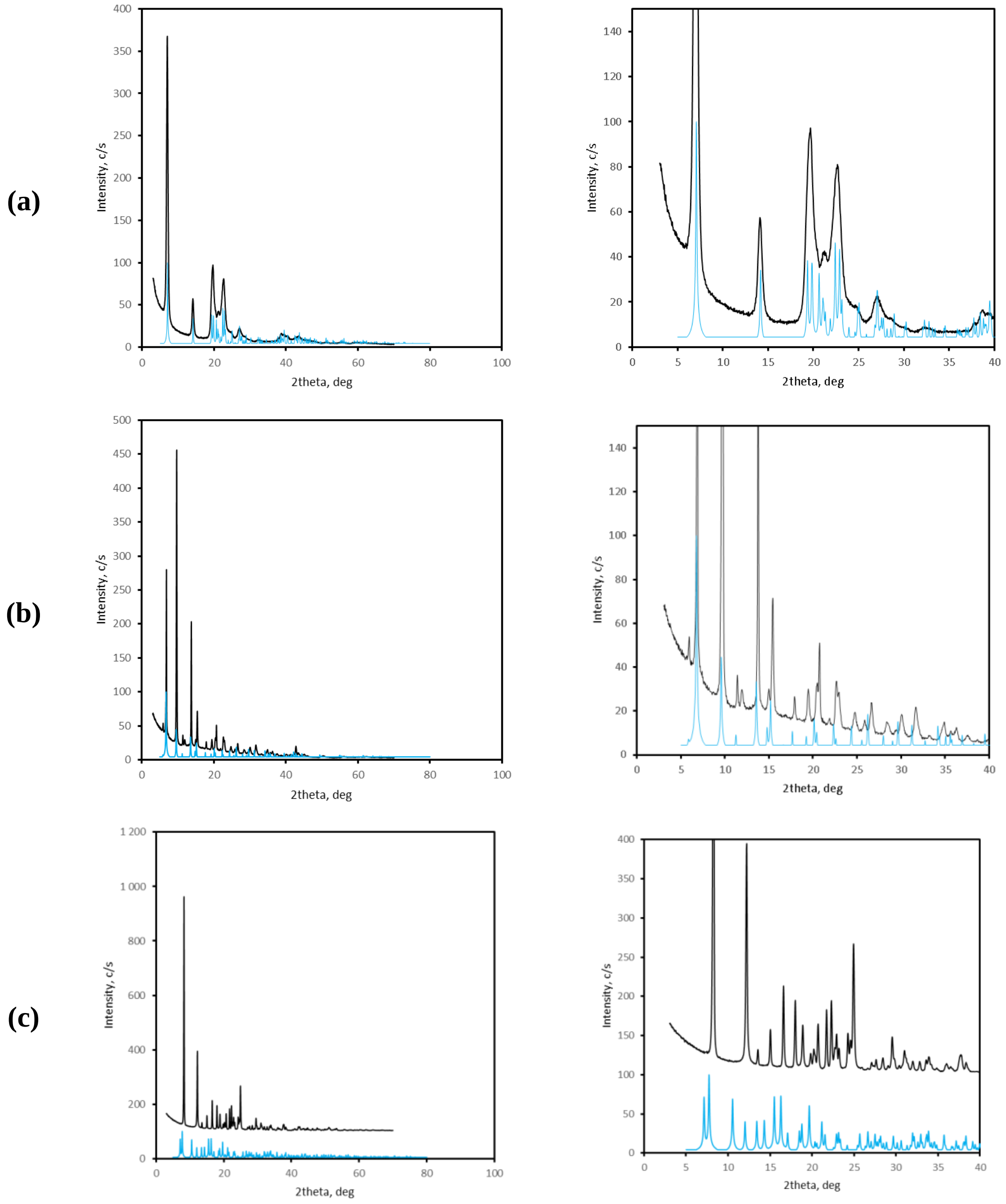

Figure S3. XRD profile of Zn-based MOFs with simulated profile overlay: (a) Zn(SEB) (black line) and poly[([mu]4-decanedio-ato)cobalt(II)] (blue line) [5], (b) Zn(BDC)Si (black line) and zinc terephthalate (Zn(BDC), MOF-5) (blue line) [6], and (c) Zn(BIM) (black line) and (Co)ZIF-9 (blue line) [7].

According to X-ray diffraction (XRD) analysis, the resulting Zn(SEB) and Zn(BDC)Si samples are single-phase (Figure S3a,b). The comparison of the diffraction pattern of Zn(SEB) was carried out by analogy with the profile simulated for cobalt sebacate (Figure S3b) as there were no cif-files in the Cambridge structure database for the resulting Zn(SEB). It was shown the identity of the structure (space group I2/a), in which the zinc atom was in the octahedral environment of the oxygens of the carboxyl groups of sebacic acid (Figure S4a). The comparison of the diffraction pattern of Zn(BDC)Si with the profile simulated for zinc terephthalate

(Zn(BDC), MOF-5) showed the identity of the structure (space group Fm3m), in which the zinc atom was in the octahedral environment of the oxygens of the carboxyl groups of silymethylated terephthalic acid (Figure S4b). The difference in porosity of Zn(BDC)Si (the presence of high microporosity and nanoporosity also) from MOF-5 (Zn(BDC)) with unsubstituted terephthalic acid is clearly associated with the orientation of the trimethylsilyl groups inside the MOF pores. The structure of the resulting Zn(BIM) was determined by comparison with a pure phase (Co)ZIF-9 [7,8]. The shift of the peaks of the XRD pattern of Zn(BIM) compared to Co-ZIF-9 XRD pattern (Figure S3c) was associated with different unit cell parameters due to the difference in the ionic radii of zinc and cobalt. The structure of Zn(BIM) was defined as R-3 (Figure S4c).

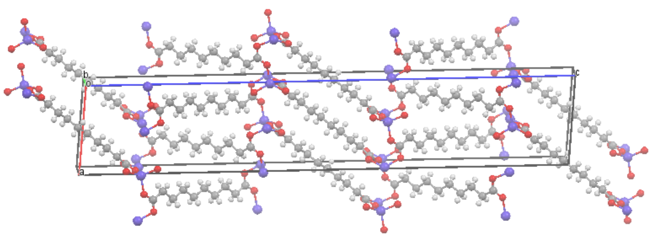

(a)

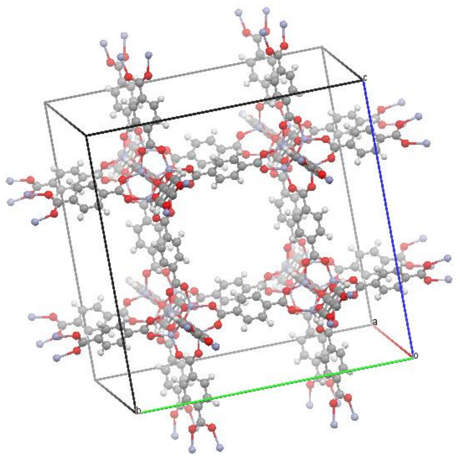

(b)

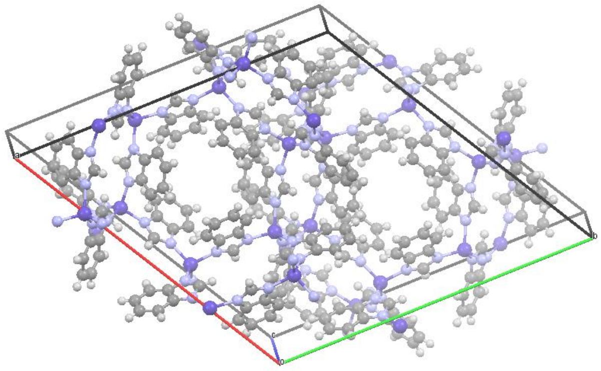

(c)

Figure S4. Structures of (a) Zn(SEB), (b) Zn(BDC)Si, (c) Zn(BIM).

The morphology of Zn-based MOFs was studied by SEM (Figures S5). The scanning electron microscopy (SEM) was carried out on a HITACHI S3400N electron microscope (Japan). The following parameters were used: exposure of 64 frames, an accelerating voltage of 20 kV, and a working distance of 10 mm.

Zn(SEB) Zn(BDC)Si Zn(BIM)

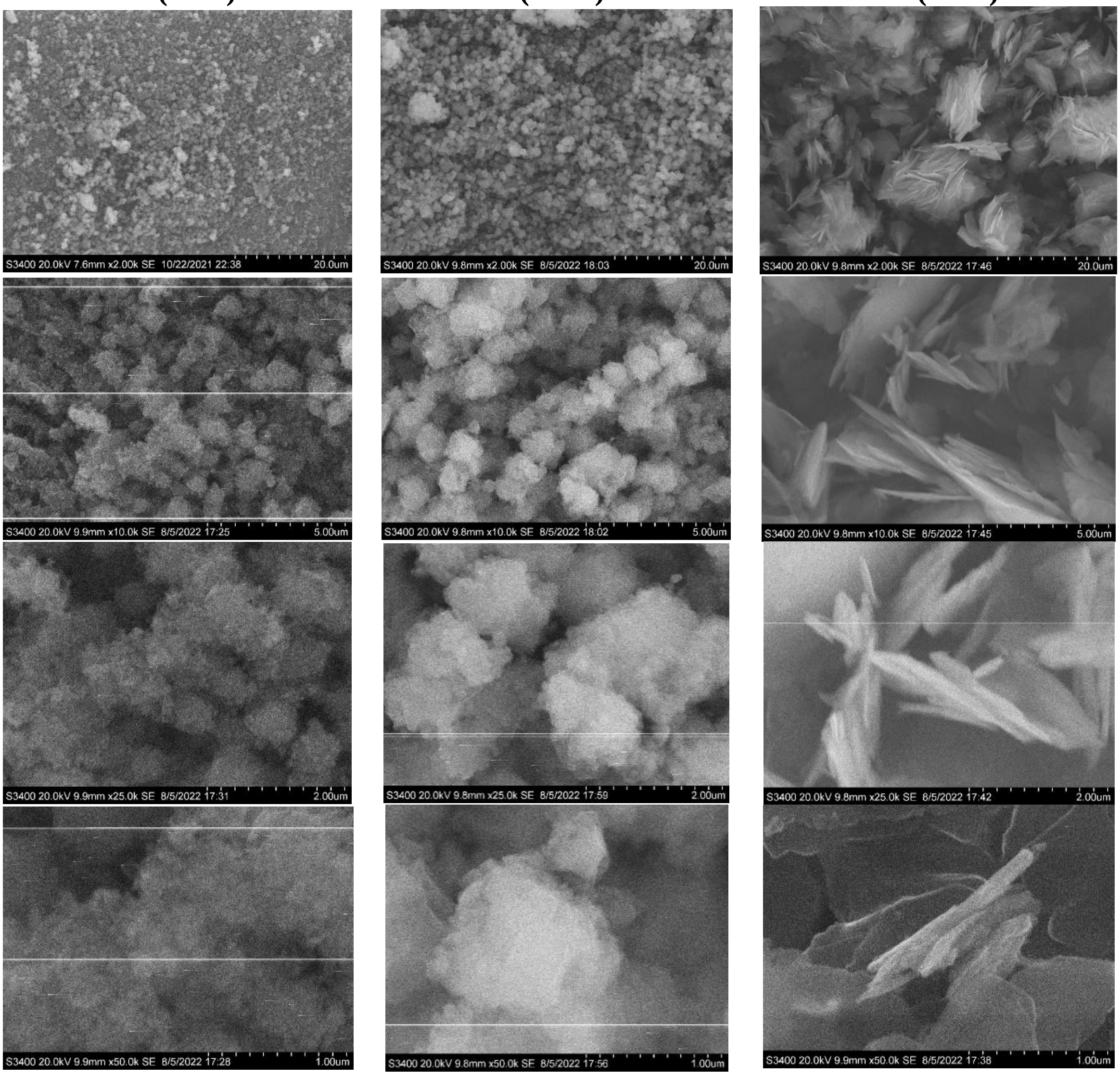

Figure S5. SEM micrographs at different magnifications of Zn-based MOFs.

It was demonstrated that Zn-based MOFs had particles of various shapes. The Zn(BDC)Si was characterized by the biggest size of particles, while the particles of Zn(BIM) had needle-shaped structure. Also, the habit of the crystals in the SEM micrographs of Zn(BIM) confirms the low-symmetry structure of the resulting compound. The smallest size of Zn(SEB) particles and their shape will contribute to a better dispersion of this MOF in the polymer matrix [9].

S2. The characterization of the developed membranes

The structure of the developed membranes was investigated by X-ray diffraction (XRD) analysis using Bruker D8 DISCOVER diffractometer (Bruker, Germany) at room temperature. CuKα radiation was used. The XRD profiles of the porous CA-12 and dense CMC membranes modified with Zn(SEB), Zn(BDC)Si and Zn(BIM) were compared with the XRD profiles of synthesized Zn-based MOFs to determine the structural integrity of modifiers in membranes. Shifted diffractograms were presented in Figure S6 for ease of analysis.

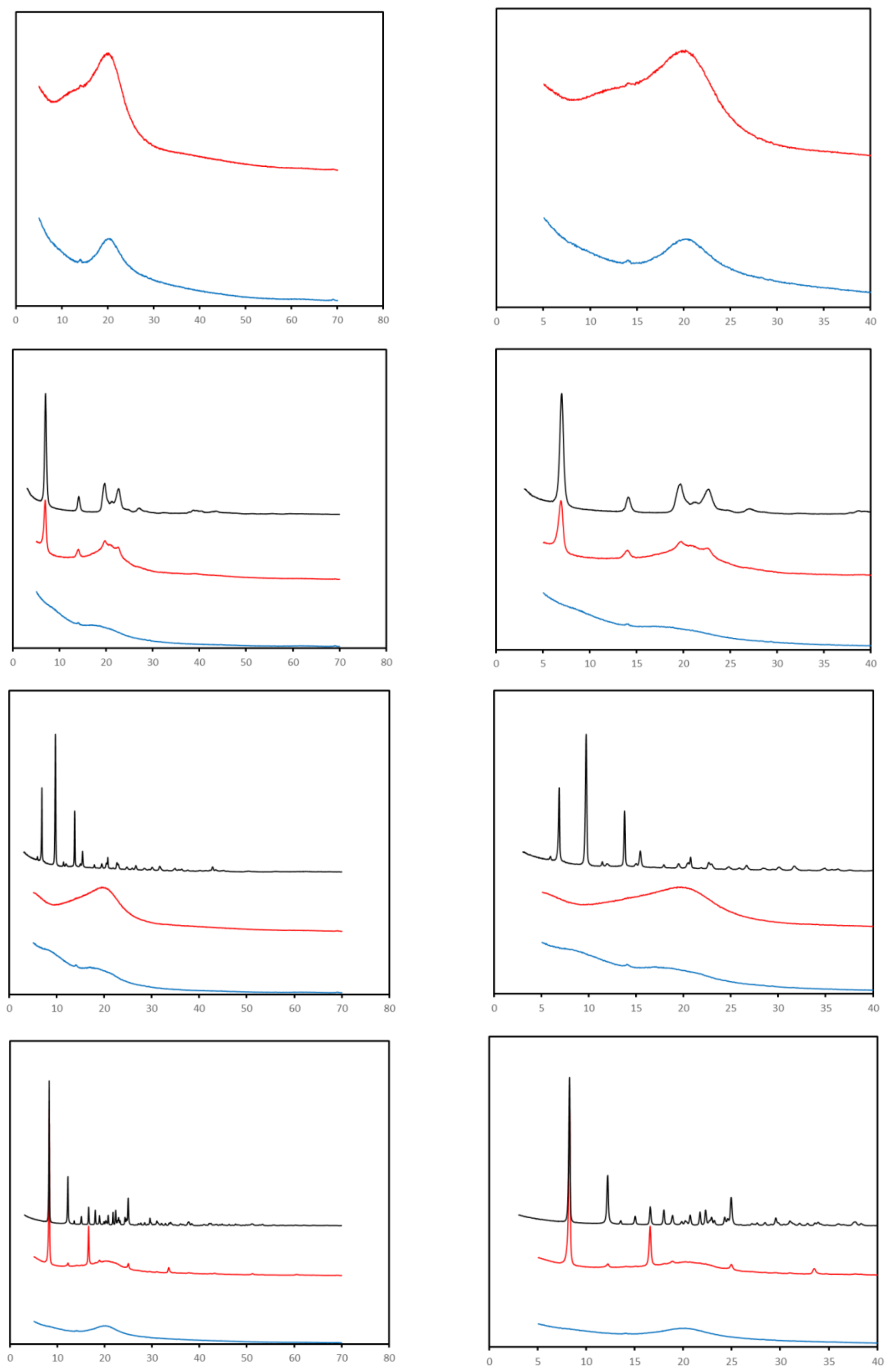

Figure S6. Shifted XRD profiles of (a) the porous CA-12 (blue line) and dense CMC (red line) membranes, (b) the modified with Zn(SEB) porous CA-12 (blue line) and dense CMC (red line) membranes and Zn(SEB) (black line), (c) the modified with Zn(BDC)Si porous CA-12 (blue line) and dense CMC (red line) membranes and Zn(BDC)Si (black line), and (d) the modified with Zn(BIM) porous CA-12 (blue line) and dense CMC (red line) membranes and Zn(BIM) (black line).

The amorphous state was observed for pristine CA-12, CMC membranes (Figure S6a) and CA-12 membranes containing a small amount (1 wt.%) of Zn-based MOFs (blue lines in

Figure S6 b,c,d). For CMC membranes containing 15 wt.% Zn(SEB) and Zn(BIM), the main peaks of the Zn-based MOF phase appeared above the halo of the amorphous phase without changes (red lines in Figure S6 b,d). This may indicate the absence of a noticeable distortion or change in the MOF structure. The absence of characteristic Zn(BDC)Si peaks in the XRD profile of CMC/Zn(BDC)Si (15 wt.%) membrane may be explained by lower modifier volume fraction compared to others, associated with a higher density of this MOF.

The stability and reusability of the developed modified membranes with improved properties (porous CA-12/Zn(SEB) (1%) and supported cross-linked CMC+Zn(BDC)Si (15%)/CA-15 membranes) were studied in nanofiltration of water and a model solution of heavy metal ions (Cd^{2+}, Pb^{2+}, Cu^{2+}) for 6 days (Figure S7). The nanofiltration experiment was as follows: during each day, the membrane was tested with water and a model solution of heavy metal ions (Cd^{2+}, Pb^{2+}, Cu^{2+}), alternating them, the membrane was taken out of the installation at night, and the experiment was continued the next day under the same conditions. This continued for 6 days. Each day's data obtained was averaged. The pristine porous CA-12 and supported cross-linked CMC/CA-15 membranes were also tested for the comparison (Figure S7).

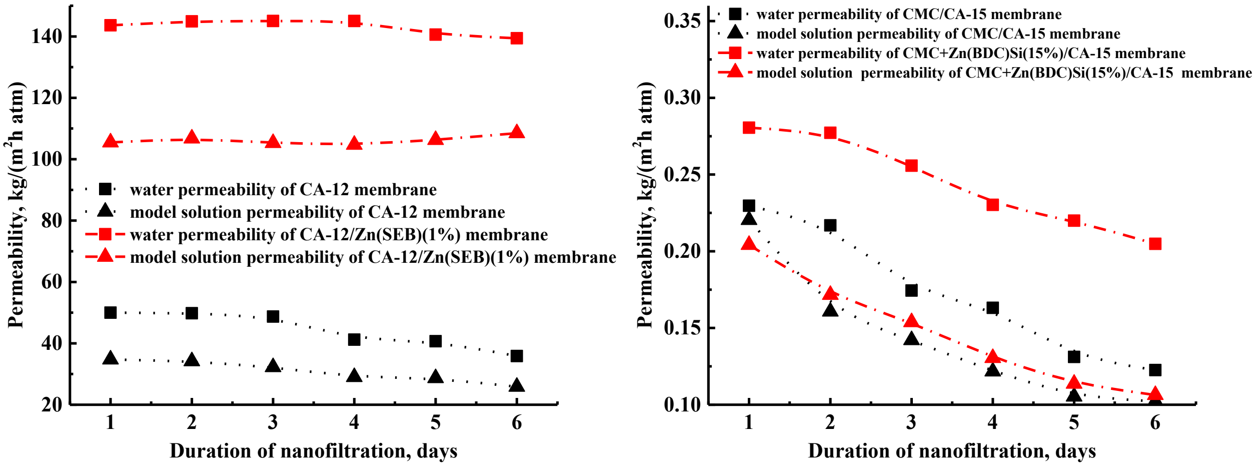

(a) (b)

Figure S7. The dependence of the permeability of water and a model solution of heavy metal ions (Cd^{2+}, Pb^{2+}, Cu^{2+}) on the number of days of nanofiltration experiments for (a) porous CA-12 and CA-12/Zn(SEB) (1%) membranes, (b) supported cross-linked CMC/CA-15 and CMC+Zn(BDC)Si (15%)/CA-15 membranes.

It was demonstrated that the permeability of the pristine CA-12 membrane slightly decreased by the 6th day of the nanofiltration experiment: from 50 to 36 kg/(m^2 h atm) for water permeability and 35 to 26 kg/(m^2 h atm) for a model solution of heavy metal ions permeability (Figure S7a). While the modified porous membrane CA-12/Zn(SEB) (1%) maintained their permeability values at the same level for 6 days of nanofiltration experiment (Figure S7a). This may be due to the lower surface roughness and higher surface hydrophilicity of the modified membrane, which contributes to less clogging of the membrane porous structure maintaining the same level of permeability. The permeability of water and a model solution of heavy metal ions (Cd^{2+}, Pb^{2+}, Cu^{2+}) during 6 days of nanofiltration decreased for the supported cross-linked CMC/CA-15 and CMC+Zn(BDC)Si (15%)/CA-15 membranes (Figure S7b). But the permeability of the modified membrane decreased to a lesser extent compared to a pristine membrane due to structural and physicochemical changes during modification with Zn(BDC)Si. The rejection coefficient values for all membranes for 6 days slightly varied by no more than

5%. The data obtained confirm the possibility of using the developed membranes repeatedly and for a long time.

S3. The characterization of the developed substrates

It is known that a porous membrane used as a substrate of supported membranes has a significant effect on the mass transfer of substances during membrane separation, in particular, its polymer type, the porosity, surface roughness, etc. [10]. In this section, to explain the nanofiltration mass transfer mechanism for cross-linked supported CMC-based membranes, developed porous CA-15, PA-15 and PAN-15 membranes used as substrates were studied by scanning electron (SEM) and atomic force (AFM) microscopies, the standard porosimetry method, and contact angle measurements. The SEM, AFM, contact angle and porosity data for the porous CA-15 membrane presented in the main text of the manuscript were also repeated for the convenience of comparing the obtained characteristics of substrates. The cross-sectional morphology and surface topography of CA-15, PA-15 and PAN-15 membranes were investigated by SEM and AFM (Figure S8).

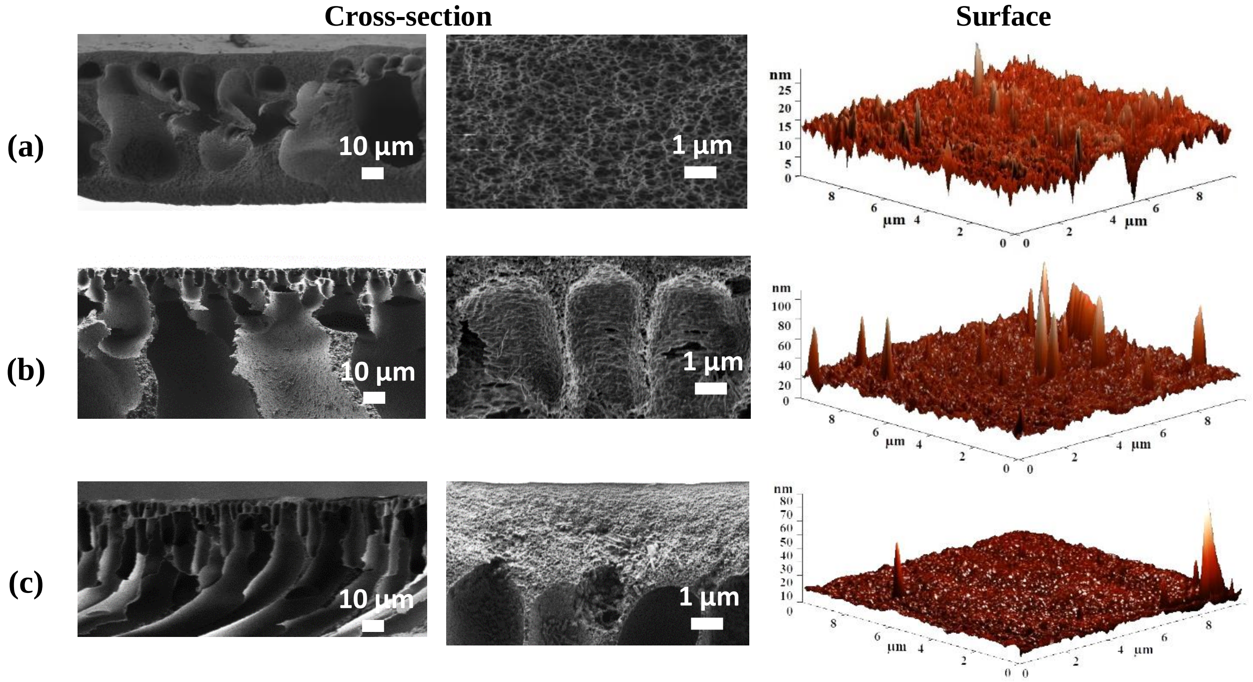

Figure S8. Cross-sectional SEM micrographs at different magnifications and surface AFM images of (a) CA-15, (b) PA-15 and (c) PAN-15 substrates.

It was demonstrated that the cross-sectional structure of these substrates prepared by the same phase inversion method via a non-solvent induced phase separation (NIPS) technique [11,12] was significantly different. There is the form of vacuole-shaped pore structure for CA-15 substrate and “finger-shaped” inner pores for PA-15 and PAN -15 substrates. It should be noted that for the PA-15 substrate, the thinnest dense top layer was observed compared to the others. The CA-15 substrate had the highest porosity of the substructure, while for PAN-15 substrate the density of the porous structure was the highest.

Based on the AFM images, the average surface roughness (Ra) of the substrates was calculated (Table S1). To assess the hydrophilic-hydrophobic balance of the substrate surface, the contact angles were measured by attached bubble method [13] using a Goniometer LK-1 (OOO NPK Open Science, Krasnogorsk, Russia), and the total porosity of the membranes was also evaluated by the standard porosimetry method using a Porosimeter 3.1 instrument (POROTECH Ltd., Woodbridge, ON, Canada) [11]. The data obtained are presented in Table S1.

Table S1. The average surface roughness, contact angle and total porosity for the developed porous substrates.

Porous membrane (substrate)	Ra, nm	Contact angle, °	Total porosity, %
CA-15	1.9	31 ± 2	94.4
PA-15	3.6	34 ± 2	91.4
PAN-15	1.2	36 ± 2	91.2

It was shown that the most surface roughness was possessed by the PA-12 substrate. The CA-12 substrate was characterized by the most hydrophilic surface (the contact angle of 31°) and the highest total porosity value due to the high porous inner structure (confirmed by the SEM data in Figure S8). While the PAN-15 substrate had the lowest surface roughness and the most hydrophobic surface (the contact angle of 36°). The contact angle data obtained for the PA-15 and PAN-15 substrates consistent with previous studies [11,12]. The different nature of polymers, porous structure, surface roughness and hydrophilicity greatly affect the transport properties of the supported CMC-based membranes prepared using these substrates.

S4. Comparison of membrane performance in nanofiltration of heavy metal ions

The performance of the developed modified membranes with improved properties (porous CA-12/Zn(SEB) (1%) and supported cross-linked CMC+Zn(BDC)Si (15%)/CA-15 membranes) was compared with described in the literature membranes for nanofiltration of heavy metal ions in terms of permeability and rejection coefficients (Table S2 and S3).

Table S2. Comparison of transport properties for the porous CA-based membranes in nanofiltration of heavy metal ions.

Membrane	Water permeability, kg/(m^{2}h atm)	Aqueous solution, concentration	Solution permeability	Rejection coefficient, %				Ref.
				Cd^{2+}	Pb^{2+}	Cu^{2+}	Co^{2+}	
CA-12/Zn(SEB) (1%)	142	$Cu(NO_3)_2$, $Pb(NO_3)_2$, $Cd(NO_3)_2$ 50 mg/L of each salt	105	63	78	47	-	This study
		$Cu(NO_3)_2$ 100 mg/L	127	-	-	64	-	
CA-22	10	$Cd(NO_3)_2$ 10^{-3} mol/L	-	80	-	-	-	[14]
CA-25	7		-	90	-	-	-	
CA	4	$CuSO_4$ 1000 ppm	3	-	-	51	-	[15]
		$CoSO_4$ 1000 ppm	2.5	-	-	-	45	
CA/MOF-5 (0.5%)	7	$CuSO_4$ 1000 ppm	6	-	-	53	-	
		$CoSO_4$ 1000 ppm	5.7	-	-	-	77	

It was demonstrated that the developed porous CA-12/Zn(SEB) (1%) membrane had improved permeability of water and heavy metal ions solution with high level of rejection coefficients compared to the CA-based membranes described in the literature. A significant difference in the performance of the developed membrane from those presented can be due to the low concentration of the casting solution (12%). It should be also noted that there are no studies of the CA-based membranes in the nanofiltration of solutions of a heavy metal ions mixture.

Table S3. Comparison of transport properties for the nonporous biopolymer membranes in nanofiltration of heavy metal ions.

**Membrane**	**Water permeability, kg/(m²h atm)**	**Aqueous solution, concentration**	**Solution permeability**	**Rejection coefficient, %**				**Ref.**
				**Cd^{2+}**	**Pb^{2+}**	**Cu^{2+}**	**Cr^{2+}**	
CMC+Zn(BDC)Si (15%)/CA-15	0.28	$Cu(NO_3)_2$, $Pb(NO_3)_2$, $Cd(NO_3)_2$ 50 mg/L of each salt	0.2	94	97	97	-	This study
nanocellulose (BNC)/poly-dopamine (PDA)	82.5	Pb(II) 58 ppm	-	-	40	-	-	[16]
		Cd(II) 50 ppm		30	-	-	-	
graphene oxide/chitosan nanoplates (1%) incorporated into PES	9.23	$CrSO_4$ 0.01 mol/L	-	-	-	-	75	[17]
CA/vinyl triethoxysilane modified graphene oxide (GO) (3%)	-	$Pb(NO_3)_2$ 20 mg/L	6.2	-	52	-	-	[18]
CA/vinyl triethoxysilane modified graphene oxide (GO) (3%)/gum Arabic (GuA) (8%)	-		8.6	-	98	-	-	

The performance comparison of the nonporous biopolymer membranes in nanofiltration of heavy metal ions showed that the developed supported cross-linked CMC+Zn(BDC)Si (15%)/CA-15 membrane exhibited lower permeability compared to others, but improved rejection coefficients of heavy metal ions. It should be also emphasized that there are no studies of the CMC-based membranes in the nanofiltration of solutions of a heavy metal ions mixture. Thus, the developed modified membranes with improved properties (porous CA-12/Zn(SEB) (1%) and supported cross-linked CMC+Zn(BDC)Si (15%)/CA-15 membranes) demonstrated promising application in the industry for water treatment from heavy metal ions depending on the separation tasks.

References

1. Choi, K.-Y.; Hie, M.Y.; Choi, S.-K. Synthesis and characterization of aromatic polymers containing pendant silyl groups. I. Polyarylates. *J. Polym. Sci. Part A Polym. Chem.* **1992**, *30*, 1575–1581, doi:10.1002/pola.1992.080300809.
2. Brunauer, S.; Emmett, P.H.; Teller, E. Adsorption of Gases in Multimolecular Layers. *J. Am. Chem. Soc.* **1938**, *60*, 309–319, doi:10.1021/ja01269a023.
3. Barrett, E.P.; Joyner, L.G.; Halenda, P.P. The Determination of Pore Volume and Area Distributions in Porous Substances. I. Computations from Nitrogen Isotherms. *J. Am. Chem. Soc.* **1951**, *73*, 373–380, doi:10.1021/ja01145a126.
4. Harkins, W.D.; Jura, G. Surfaces of Solids. XII. An Absolute Method for the Determination of the Area of a Finely Divided Crystalline Solid. *J. Am. Chem. Soc.* **1944**, *66*, 1362–1366, doi:10.1021/ja01236a047.
5. Giuseppe, B.; Francesco, N.; Giovanni, G.; Alessandro, S.; Mollica Nardo, V. Poly[(μ 4 -decanedioato)cobalt(II)]. *Acta Crystallogr. Sect. E Struct. Reports Online* **2014**, *70*, m159–m159, doi:10.1107/S1600536814006011.
6. Trousselet, F.; Archereau, A.; Boutin, A.; Coudert, F.-X. Heterometallic Metal–Organic Frameworks of MOF-5 and UiO-66 Families: Insight from Computational Chemistry. *J. Phys. Chem. C* **2016**, *120*, 24885–24894, doi:10.1021/acs.jpcc.6b08594.
7. Park, K.S.; Ni, Z.; Côté, A.P.; Choi, J.Y.; Huang, R.; Uribe-Romo, F.J.; Chae, H.K.;

O'Keeffe, M.; Yaghi, O.M. Exceptional chemical and thermal stability of zeolitic imidazolate frameworks. *Proc. Natl. Acad. Sci.* **2006**, *103*, 10186–10191, doi:10.1073/pnas.0602439103.
8. Öztürk, Z.; Hofmann, J.P.; Lutz, M.; Mazaj, M.; Logar, N.Z.; Weckhuysen, B.M. Controlled Synthesis of Phase-Pure Zeolitic Imidazolate Framework Co-ZIF-9. *Eur. J. Inorg. Chem.* **2015**, *2015*, 1625–1630, doi:10.1002/ejic.201403077.
9. Elrasheedy, A.; Nady, N.; Bassyouni, M.; El-Shazly, A. Metal Organic Framework Based Polymer Mixed Matrix Membranes: Review on Applications in Water Purification. *Membranes (Basel).* **2019**, *9*, 88, doi:10.3390/membranes9070088.
10. Dmitrenko, M.; Kuzminova, A.; Zolotarev, A.; Ermakov, S.; Roizard, D.; Penkova, A. Enhanced pervaporation properties of PVA-based membranes modified with polyelectrolytes. application to IPA dehydration. *Polymers (Basel).* **2020**, *12*, doi:10.3390/polym12010014.
11. Dmitrenko, M.; Kuzminova, A.; Zolotarev, A.; Liamin, V.; Plisko, T.; Burts, K.; Bildyukevich, A.; Ermakov, S.; Penkova, A. Novel High Flux Poly(m-phenylene isophtalamide)/TiO2 Membranes for Ultrafiltration with Enhanced Antifouling Performance. *Polymers (Basel).* **2021**, *13*, 2804, doi:10.3390/polym13162804.
12. Dmitrenko, M.; Kuzminova, A.; Zolotarev, A.; Markelov, D.; Komolkin, A.; Loginova, E.; Plisko, T.; Burts, K.; Bildyukevich, A.; Penkova, A. Modification strategies of polyacrylonitrile ultrafiltration membrane using TiO2 for enhanced antifouling performance in water treatment. *Sep. Purif. Technol.* **2022**, *286*, 120500, doi:10.1016/j.seppur.2022.120500.
13. Plisko, T. V.; Liubimova, A.S.; Bildyukevich, A. V.; Penkova, A. V.; Dmitrenko, M.E.; Mikhailovskii, V.Y.; Melnikova, G.B.; Semenov, K.N.; Doroshkevich, N. V.; Kuzminova, A.I. Fabrication and characterization of polyamide-fullerenol thin film nanocomposite hollow fiber membranes with enhanced antifouling performance. *J. Memb. Sci.* **2018**, *551*, 20–36, doi:10.1016/j.memsci.2018.01.015.
14. Ounifi, I.; Ursino, C.; Santoro, S.; Chekir, J.; Hafiane, A.; Figoli, A.; Ferjani, E. Cellulose acetate nanofiltration membranes for cadmium remediation. *J. Membr. Sci. Res.* **2020**, *6*, 226–234, doi:10.22079/JMSR.2020.120669.1336.
15. Gnanasekaran, G.; Balaguru, S.; Arthanareeswaran, G.; Das, D.B. Removal of hazardous material from wastewater by using metal organic framework (MOF) embedded polymeric membranes. *Sep. Sci. Technol.* **2019**, *54*, 434–446, doi:10.1080/01496395.2018.1508232.
16. Gholami Derami, H.; Jiang, Q.; Ghim, D.; Cao, S.; Chandar, Y.J.; Morrissey, J.J.; Jun, Y.-S.; Singamaneni, S. A Robust and Scalable Polydopamine/Bacterial Nanocellulose Hybrid Membrane for Efficient Wastewater Treatment. *ACS Appl. Nano Mater.* **2019**, *2*, 1092–1101, doi:10.1021/acsanm.9b00022.
17. Bagheripour, E.; Moghadassi, A.R.; Hosseini, S.M.; Van der Bruggen, B.; Parvizian, F. Novel composite graphene oxide/chitosan nanoplates incorporated into PES based nanofiltration membrane: Chromium removal and antifouling enhancement. *J. Ind. Eng. Chem.* **2018**, *62*, 311–320, doi:10.1016/j.jiec.2018.01.009.
18. Idress, H.; Zaidi, S.Z.J.; Sabir, A.; Shafiq, M.; Khan, R.U.; Harito, C.; Hassan, S.; Walsh, F.C. Cellulose acetate based Complexation-NF membranes for the removal of Pb(II) from waste water. *Sci. Rep.* **2021**, *11*, 1806, doi:10.1038/s41598-020-80384-0.
